# Combined Inhibition of PI3K and STAT3 signaling effectively inhibits bladder cancer growth

**DOI:** 10.1038/s41389-024-00529-y

**Published:** 2024-07-27

**Authors:** Weidong Peng, Haojie Zhang, Mingwei Yin, Dejie Kong, Liping Kang, Xinkun Teng, Jingjing Wang, Zhimin Chu, Yating Sun, Pengpeng Long, Chengying Cui, Bin Lyu, Jinzhi Zhang, Han Xiao, Mingqing Wu, Yongqiang Wang, Yang Li

**Affiliations:** 1 Department of Epidemiology and Health Statistics, School of Public Health, Anhui Medical University, Hefei, China; 2 Department of Genetics, School of Life Science, Anhui Medical University, Hefei, China; 3 Department of Urology, Huadong Hospital, Fudan University, Shanghai, China; 4 Department of Pathophysiology, School of Basic Medical Sciences, Anhui Medical University, Hefei, China; 5 Department of Pathology, The First Affiliated Hospital of Anhui Medical University, Hefei, China; 6 Department of Urology, South China Hospital, Medical School, Shenzhen University, Shenzhen, China; 7 Anhui Province Key Laboratory of Urological and Andrological Diseases Research and Medical Transformation, Hefei, China

**Keywords:** Targeted therapies, Urological cancer

## Abstract

Bladder cancer is characterized by aberrant activation of the phosphatidylinositol-3-OH kinase (PI3K) signaling, underscoring the significance of directing therapeutic efforts toward the PI3K pathway as a promising strategy. In this study, we discovered that PI3K serves as a potent therapeutic target for bladder cancer through a high-throughput screening of inhibitory molecules. The PI3K inhibitor demonstrated a robust anti-tumor efficacy, validated both in vitro and in vivo settings. Nevertheless, the feedback activation of JAK1-STAT3 signaling reinstated cell and organoid survival, leading to resistance against the PI3K inhibitor. Mechanistically, the PI3K inhibitor suppresses PTPN11 expression, a negative regulator of the JAK-STAT pathway, thereby activating STAT3. Conversely, restoration of PTPN11 enhances the sensitivity of cancer cells to the PI3K inhibitor. Simultaneous inhibition of both PI3K and STAT3 with small-molecule inhibitors resulted in sustained tumor regression in patient-derived bladder cancer xenografts. These findings advocate for a combinational therapeutic approach targeting both PI3K and STAT3 pathways to achieve enduring cancer eradication in vitro and in vivo, underscoring their promising therapeutic efficacy for treating bladder cancer.

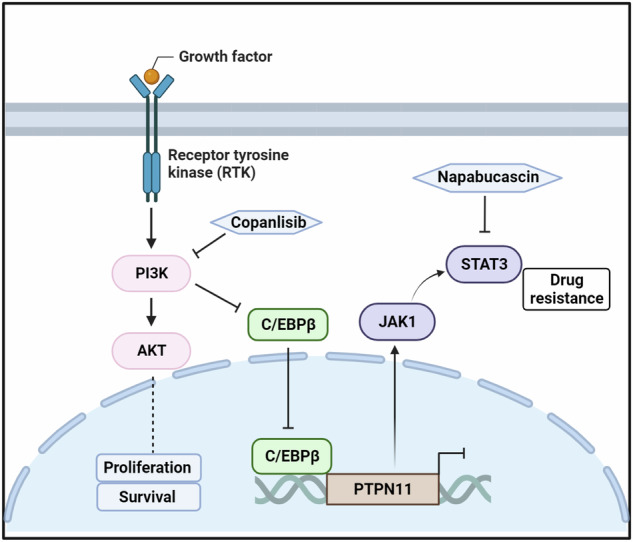

## Introduction

Bladder cancer (BCa) is one of the most commonly diagnosed cancers worldwide and is the fourth most common malignancy in men [1]. Over the past 10 years, significant advances in treatment have been made despite there are still more than 200,000 deaths caused by BCa per year globally [2], mainly owing to approximately 50% of patients will develop recurrence, metastatic, and drug resistance, and patients at an advanced or metastatic stage are generally considered incurable [3]. Therefore, there is an unmet demand to discover novel strategies to treat advanced BCa.

To date, the primary treatments available for advanced BCa besides cystectomy include chemotherapy, immune checkpoint inhibitors (ICIs), and targeted therapies [4]. Unfortunately, chemotherapy is fraught with inevitable drug resistance and toxicity concerns [5], and ICIs achieve durable responses only in a small subset of patients (15%-20%) [6]. Erdafitinib is the only approved molecule by the United States Food and Drug Administration (FDA) for BCa treatment, which is applicable for advanced BCa with susceptible FGFR3 alterations and provides modest effects, conferring a response rate of 40% but median progression-free only 2.8 months [7, 8].

In the past decade, targeted therapies progressed rapidly and have profoundly benefited many patients with solid tumor [9]. For instance, trastuzumab, which targets the HER2 receptor, has dramatically improved overall survival (OS) to 37% in women with HER2-positive breast cancer [10]. In non-small cell lung cancer (NSCLC), patients who harbored EGFR mutations treated with gefitinib achieved a response rate of 75%, with a median progression-free of 7-12 months [11]. However, the exploration of targeted therapy for BCa remains in the beginning stage of research [12]. As a common malignancy with high tumor mutation burden, advanced BCa is likely to respond to proper small molecular inhibitors, relying on deep insights into the molecular features of BCa. According to The Cancer Genome Atlas (TCGA), BCa harbors a high mutational load; the most leading alteration is PIK3CA with a ratio of approximately 25%, following by 20% FGFR2 or FGFR3 activating alterations which are the targets of erdafitinib [13]. The integrated analysis also identified mutation and copy-number data, which revealed that the phosphatidylinositol-3-OH kinase (PI3K) pathway was altered in 72% of the BCa [14]. PI3K/AKT is a well-known oncogenic pathway related to cell proliferation, migration, and invasion in a wide context of cancer, including BCa [14–16]. Moreover, it also functions downstream of FGFR2/3 in BCa tumorigenesis [17]. Therefore, downregulating the activity of the PI3K pathway emerges as a promising strategy for BCa treatment.

PI3K inhibitors, such as copanlisib and idelalisib [16], have shown promising therapeutic efficacy and have been clinically approved for use in several tumor types such as lymphoma and breast cancer [18, 19], and also demonstrated certain clinical activity in BCa therapy. In a phase II trial, seven out of thirteen patients with metastatic urothelial carcinoma demonstrated a response to PI3K inhibitor buparlisib, with an objective remission rate (ORR) of 53.84%, particularly for patients with TSC1 or ERBB2 mutation, along with AREG/EREG gene amplification [20], which suggested that besides patients bearing PI3K mutations, patients with activated PI3K signaling will also benefit from PI3K inhibitors. However, PI3K inhibitors have not been approved by the FDA as monotherapy for patients with solid tumors so far, mainly due to concomitant MAPK or other bypass signaling pathway activation, reducing dependence on PI3K signaling for tumor growth and proliferation [21]. Therefore, a systematic investigation of the effect and underlying molecular changes upon PI3K blockade in robust preclinical models of BCa will provide clues of combinational therapy to overcome drug resistance and improve patient survival outcomes.

In the current study, we identified PI3K as an effective therapeutic target for BCa through high-throughput inhibitory molecules screening, and a prompt and effective anti-tumor efficacy of PI3K inhibitor was verified both in vitro and in vivo. However, drug resistance will develop inevitably after long-term treatment with PI3K inhibitor. RNA sequencing (RNA-seq) analysis along with the Phospho-RTK Array indicated that feedback activation of JAK1-STAT3 signaling after PI3K blockade facilitated cell survival and caused treatment failure, mainly through the inhibition of PTPN11 expression. Combined administration of both PI3K and STAT3 inhibitors achieved a long-term cancer elimination in vitro and in vivo, highlighting their therapeutic potential for BCa treatment.

## Materials and methods

### Clinical samples and cell lines

The human samples were obtained from the First Affiliated Hospital of Anhui Medical University, 63 pairs of BCa tissues, and matched para-tumor tissues (PT) from the same patient were collected with written consent before enrollment. BCa cell lines: 5637 (ATCC NO. HTB-9), UM-UC-3 (ATCC NO. CRL-1749), J82 (ATCC NO. HTB-1), and T24 (ATCC NO. HTB-4), SW780 (ATCC NO. CRL-2169), TCCSUP (ATCC NO. HTB-5), as well as the immortalized uroepithelial cell line SV-HUC-1 (ATCC NO. CRL-9520) were purchased in 2018 from the Chinese Academy of Cell Resource Center (Shanghai, China), where they were authenticated with short tandem repeat profiling. Cells were maintained as previously described [22]. All the cells were authenticated by STR profiling and were commonly tested for mycoplasma contamination and not cultured for longer than 20 passages (Supplementary Table S1).

### Mouse models

The N-butyl-N-4-hydroxybutyl Nitrosamine (BBN)-induced bladder carcinogenesis mice model was established as previously described [23]. Briefly, Male C57BL/6 J mice (6 weeks old) were given drinking water containing 0.05% (w/v) BBN (TCI, catalog no. B0938) for 20 weeks. After the BBN administration, mice were given normal drinking water and were randomized into 4 groups (n = 11) for treatment with DMSO (as a control), (10 mg/kg) copanlisib (MCE BAY 80-6946), (4 mg/kg) napabucasin (MCE BBI608), or both drugs (10 mg/kg copanlisib and 4 mg/kg napabucasin) i.p. every 3 days. After seven injections, mice were euthanized for tissue retrieval, and bladders of treated mice were collected for HE and immunohistochemical staining (IHC) staining.

The BBN-induced bladder carcinogenesis mice model was established as described above. Mice were with DMSO (as a control), (10 mg/kg) copanlisib (MCE BAY 80-6946) i.p. every 3 days respectively. Tumor burdens were recorded through computed tomography scanning. CT images (recorded by Raycision IMAGING 100) were analyzed blindly. After eight injections, mice were euthanized for tissue retrieval.

Patient-derived xenografts of BCa were given generously by Dr Yongqiang Wang (South China Hospital Affiliated to Shenzhen University, China), tissues were cut into small pieces and implanted subcutaneously into the flanks of male athymic nude mice (6-8 weeks old). When tumors reached (300 mm^3^), mice were randomized into 4 groups (n = 4) for treatment with DMSO (as a control), (10 mg/kg) copanlisib, (4 mg/kg) napabucasin, or both drugs (10 mg/kg copanlisib and 4 mg/kg napabucasin) i.p. every 3 days. After four injections, mice were euthanized for tissue retrieval.

Orthotopic MB49 bladder tumors were generated by injection with fLuc-MB49 cells into the bladder wall of male C57BL/6 J mice following the reported protocols [24], then mice were randomized into 2 groups (n = 2) for treatment with DMSO (as a control), copanlisib (10 mg/kg) i.p. every 3 days. Mice were intraperitoneally injected with D-Luciferin potassium salt (150 mg/kg) and then imaged by the IVIS Lumina system imaging after the weekly treatment.

### Statistical analysis

All experiments were carried out three times. Statistical analyses were performed using GraphPad Prism software version 8. The correlations between phosphorylated PI3K/AKT expression and the clinicopathological characteristics of BCa were tested by performing chi-square tests. Further, survival curves were constructed using the Kaplan–Meier method and the Student’s t test was performed to compare differences between two groups of quantitative variables. Data were presented as means ± SD or number (percentage), and statistical significance was set at *P* < 0.05.

Detailed materials and methods are available in Supplementary Materials and Methods.

## Results

### PI3K is an effective therapeutic target for BCa

**Fig. 1:**
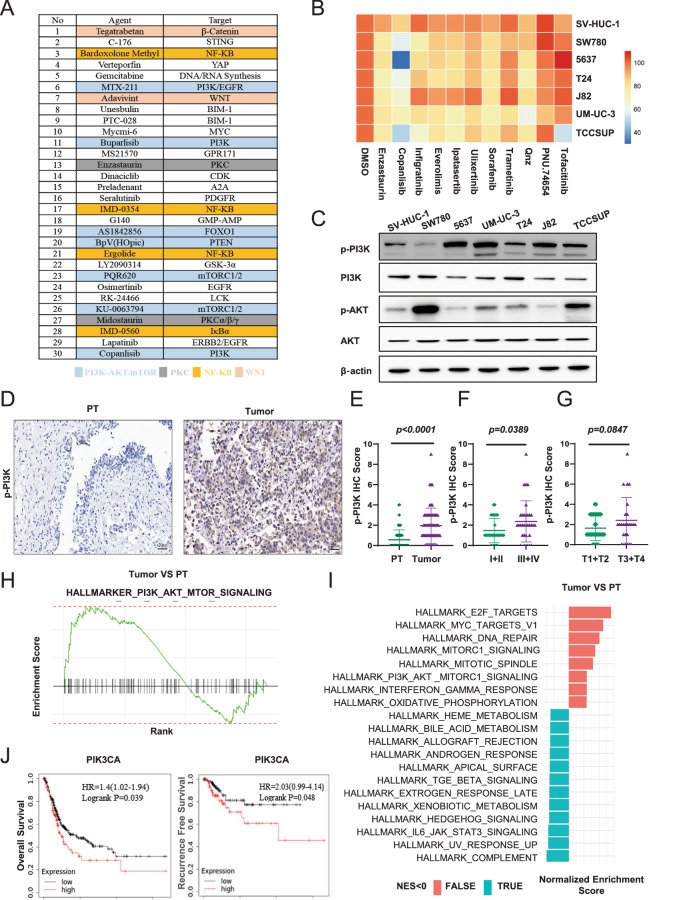
PI3K signaling is activated in BCa. **A** Top 30 compounds that induced growth inhibition of 5637 cell. Blue, PIK3/AKT/mTOR inhibitors; Gray, PKC inhibitors; Orange, NF-KB inhibitors; Pink, WNT inhibitors. **B** Heatmap showed growth inhibition of six bladder cancer cells (5637, J82, UM-UC-3, T24, SW780 and TCCSUP) and one normal urothelial cell line (SV-HUC-1) by indicated pathway inhibitors. **C** Western blot analysis of phosphorylated and total protein levels of PI3K and AKT in six bladder cancer cells and one normal urothelial cell line. β-actin served as loading controls. **D** Representative images of phosphorylated PI3K IHC staining in clinical BCa samples (n = 63); PT, para-tumor tissues. Scale bars: (20 μm). **E** IHC score of phosphorylated PI3K in tumor and matched para-tumor tissues. **F** Phosphorylated PI3K expression was analyzed based on the clinical stage. **G** Phosphorylated PI3K expression was analyzed based on the tumor invasion. **H** GSEA pathway analysis showed PI3K-ATK-mTOR pathway was significantly enriched in the GEO BCa database with tumor tissue, relative to para-tumor tissues. **I** GSEA pathway analysis exhibited differential gene pathway enrichment in the TCGA BCa datasets with tumor tissue, compared to para-tumor tissues. **J** Kaplan-Meier analysis of overall survival and recurrence free survival in BCa patients from TCGA based on the expression of PIK3CA mRNA. Red, high PIK3CA mRNA; Black, low PIK3CA mRNA (n = 405). Data are presented as mean ± SD of independent samples with individual data points shown; *P* values were assessed by two-tailed Student t test in comparison with para-tumor tissues group (**E**–**G**).

To find the effective small molecules which could be applied for BCa treatment, we first performed an HTS using a custom compounds library consisting of 150 signaling inhibitors to treat BCa cell line 5637 with the same concentration. The top 30 hit compounds that induced growth inhibition could mainly be classified into four distinct classes, including seven PI3K-AKT-mTOR inhibitors, four NF-kB inhibitors, two Wnt/β-actin inhibitors and two PKC inhibitors (Fig. 1A). To further validate the efficacy of inhibitors screened from the HTS assay, especially their potential for a broad clinical application, we performed the cell viability assay by treating six BCa cell lines (5637, UM-UC-3, J82, T24, SW780, TCCSUP) and a normal urothelial cell line SV-HUC-1 with 11 inhibitors including the above identified inhibitors. Notably, of all these tested inhibitors, PI3K inhibitor copanlisib exhibited the most inhibitory effect on all BCa cell lines but a relative mild effect on normal cell line. (Fig. 1B). Additionally, we calculated the differences in inhibition rates of various small molecule inhibitors between tumor cells and normal cells. It is evident that copanlisib still exhibits the strongest relative inhibition rate (Supplementary Fig. S1A). Besides, we examined the level of phosphorylated and total protein of PI3K and AKT in this panel of BCa cells, the results demonstrated that the activation of the PI3K pathway is a common event in most BCa cells relative to normal urothelial cells (Fig. 1C).

For clinical relevance, we performed IHC to determine the phosphorylated PI3K and AKT levels in human BCa samples (n = 63, Supplementary Table S2). Consistent with our in vitro data, it revealed that PI3K and AKT were widely phosphorylated in human BCa tissues whereas the paired PT showed barely phosphorylated staining (*P* < *0.0001*, Fig. 1D, E, Supplementary Fig. S1B). Moreover, an elevated expression of phosphorylated PI3K and AKT expression was found to be associated with worse clinical stage progression (*P* = *0.0389*, Fig. 1F and Supplementary Fig. S1C), the phosphorylated PI3K and AKT expression also demonstrated a correlation with tumor invasion, with a *p*-value close to 0.05 (*P* = *0.0847*, Fig. 1G, Supplementary Fig. S1D and Supplementary Table S3).

Additionally, the enrichment of the PI3K pathway was evident in tumor compared to PT samples in the cohort of BCa patients from both the GEO dataset (combined from the two largest BCa datasets with both tumor and normal samples, GSE31189 & GSE13507, n = 223 and 92 respectively, Fig. 1H) as well as TCGA datasets (Fig. 1I). The outcomes determined by the Kaplan-Meier plotter database (https://kmplot.com/analysis/) showed that the expression of PIK3CA correlated negatively with OS and RFS (recurrence free survival) of BCa patients (Fig. 1J). Similarly, other genes related to the PI3K pathway were also associated with poorer prognosis (Supplementary Fig. S1E).

Together, these findings suggest that the activation of PI3K is widely presented in BCa, which could be an effective therapeutic target of BCa treatment.

### PI3K inhibitor exhibits a strong anti-tumor efficacy in BCa models

Given the above evidence, we then validated the anti-tumor effect of PI3K inhibitor copanlisib in different BCa models, and FGFR3 inhibitor infigratinib was used as a control since it is one of the only two FGFR3 inhibitors approved by the FDA for BCa treatment [25], which showed a better anti-tumor effect than the other one erdafitinib in our BCa cell lines as well as established organoid models (Supplementary Fig. S2A, B).

**Fig. 2:**
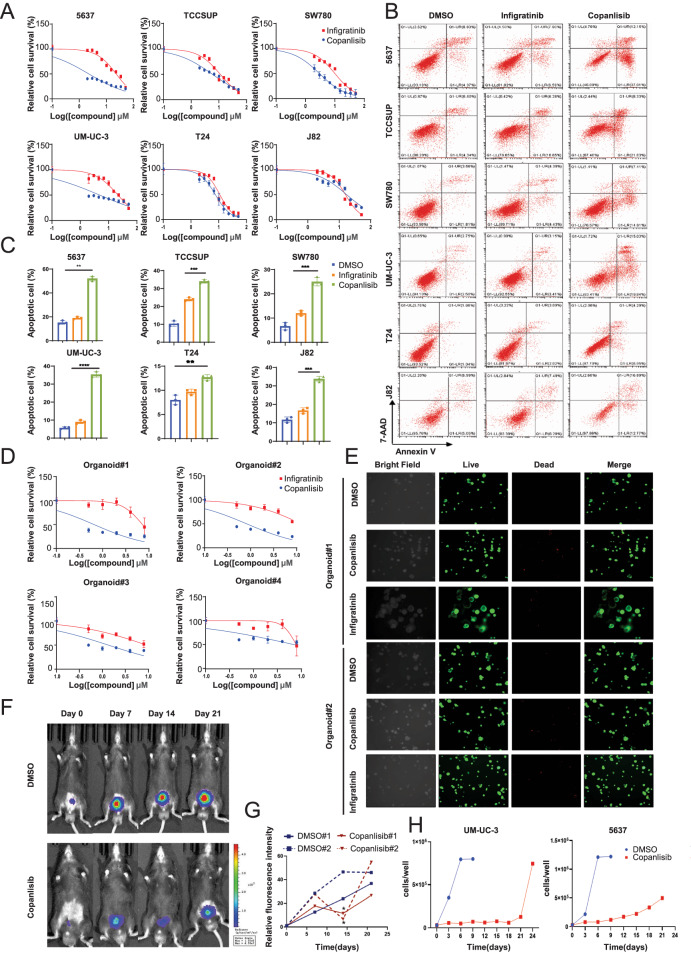
In vitro effect of PI3K inhibitor in BCa models. **A** IC_50_ assay evaluated the sensitivity of six BCa cells to infigratinib or copanlisib with a range of doses. Each point on the dose–response curves represent three technical replicates. Representative images (**B**) and quantification (**C**) of apoptosis assay in six BCa cells treated with infigratinib or copanlisib. **D** IC_50_ assay assessed the sensitivity of four organoid models to infigratinib or copanlisib with a range of doses. Each point on the dose–response curves represent three technical replicates. **E** Representative images of Live/Dead staining assay in organoid models treated with infigratinib or copanlisib. **F** Bioluminescence images of mice with MB49-fLUC tumors that received DMSO or copanlisib (representative of n = 2 mice). **G** The luminescence intensity fold change of tumor-bearing mice during DMSO or copanlisib. The stars indicate the best response of each mouse (n = 2 mice). **H** Population doublings of 5637 and UM-UC-3 cells treated with DMSO or copanlisib. Data are presented as mean ± SD of independent samples with individual data points shown; *P* values were assessed by two-tailed Student t test in comparison with infigratinib treated group (**C**); *, *P* < 0.05; **, *P* < *0.01*; ***, P < *0.001*; ****, P < *0.0001*.

We assessed the IC_50_ and employed cell apoptosis assay to estimate the anti-tumor efficacy of copanlisib in six BCa cell lines. As depicted in (Fig. 2A–C), most of the six BCa cell lines are more sensitive to copanlisib than infigratinib. We also established four mouse BCa organoids from our BBN-induced BCa tissue to evaluate the anti-tumor effect of copanlisib (Supplementary Fig. S2C) [26]. The hematoxylin-eosin (H&E) staining of paraffin sections along with bright-field images of the organoids and morphological changes after an 8-day culture were shown in Supplementary Fig. S2D, E. Organoid viability was measured by Cell-Titer Glo assay after 2 days of copanlisib and infigratinib treatment, which showed copanlisib had a stronger inhibitory effect of organoid viability than infigratinib (Fig. 2D). In addition, the organoid-specific Live/Dead staining assay also proved that copanlisib induced more BCa organoid death than infigratinib (Fig. 2E and Supplementary Fig. S2F).

We further examined whether copanlisib has similar strong anti-tumor capacities in vivo in an orthotopic BCa model, by injecting firefly luciferase-expressing MB49 cells into the bladders of C57BL/6 J mice. Tumor establishment was confirmed by the presence of bioluminescent signals 7 days after the cell injection. Mice were then randomly divided into two groups and received either copanlisib or DMSO treatment. The IVIS Lumina system was used to record the progression of orthotopic bladder tumors weekly after the treatment.

As shown in (Fig. 2F, G), signals of tumor treated with copanlisib were significantly reduced on the 14th day, indicating that inhibition of PI3K effectively suppressed the BCa growth in vivo. However, after prolonged treatment on the 21st day, all copanlisib-treated tumors relapsed. A similar response was obtained in the BBN-induced mice BCa model which was confirmed by micro computed tomography system (Supplementary Fig. S2G). Moreover, we performed a long-term drug treatment in vitro, 5637 and UM-UC-3 cells were treated with either DMSO or copanlisib. Consistent with the in vivo findings, the growth of BCa cells treated with copanlisib was drastically repressed at an early stage but recovered in the late stage (Fig. 2H), indicating an acquired resistance to PI3K inhibitor caused by intrinsic changes in BCa cells instead of microenvironmental interventions.

Overall, these findings suggest the broad and prompt anti-BCa effect of PI3K inhibitor copanlisib. However, drug resistance will develop during long-term treatment, which partially explains the reason of limited application of PI3K inhibitors in BCa treatment.

### JAK1-STAT3 activation is involved in acquired resistance to PI3K inhibitor

**Fig. 3:**
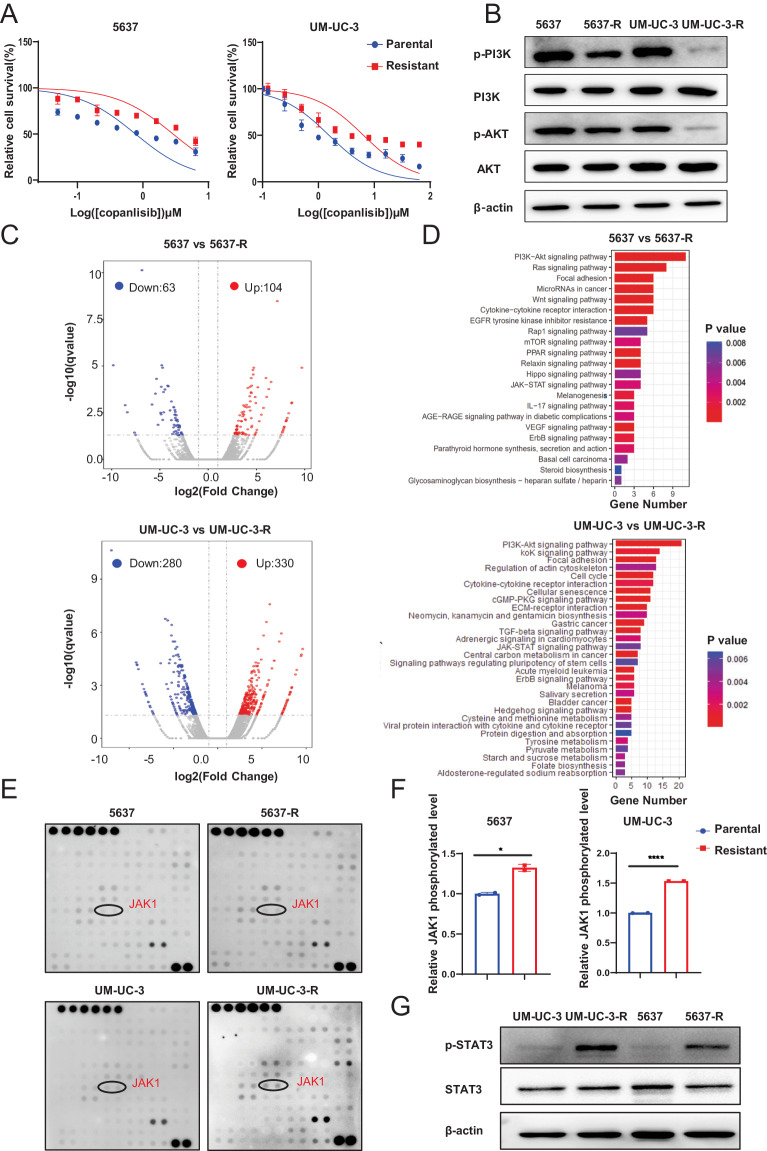
Activation of JAK1-STAT3 has been identified as a key driver of copanlisib resistance. **A** IC_50_ assay of copanlisib-resistant cell lines and parental cell lines with copanlisib treatment at indicated concentrations for 48 h. **B** Western blot analysis of phosphorylated PI3K and AKT, total PI3K and AKT protein levels in parental and resistant cancer cells. β-actin served as loading controls. **C** Volcano plot of differential expressed genes between parental and resistant cancer cells. x axis indicates the log2(Fold change), cutoff lines represent fold change > 2, and y axis shows −log10 (qvalues), cutoff line represents *P* value < 0.05, gray indicates genes with no significant differences. **D** KEGG pathways enrichment analysis of differentially expressed genes between the parental and resistant cells with a log2 fold change ≥ 1.5 and p adjust < 0.01. Representative images (**E**) and quantification (**F**) of phosphorylation signals of JAK1 in parental and copanlisib-resistant BCa cells. Cell lysates were analyzed using the Human Phospho-RTK Array Kit following the manufacturer’s instructions. Quantified phosphorylation signal was derived from two antibody spots of JAK1. **G** Western blot analysis of phosphorylated STAT3 and total STAT3 protein levels in parental and resistant cancer cells. β-actin served as loading controls. Data are presented as mean ± SD of independent samples with individual data points shown; *P* values were assessed by two-tailed Student t test in comparison with parental cells group (**F**); *, *P* < 0.05; **, *P* < *0.01*; ***, P < *0.001*; ****, P < *0.0001*.

Next, we investigated the mechanisms that involved in acquiring copanlisib resistance of BCa during long-term treatment. We used 5637 and UM-UC-3 cells to establish the acquired resistance cell lines, designated as 5637-R and UM-UC-3-R, through chronic exposure to copanlisib. The survived cells were more resistant to copanlisib than the parental cells, as demonstrated by a significant higher IC_50_ (Fig. 3A and Supplementary Fig. S3A), and the protein level of the phosphorylated PI3K and AKT remained effectively suppressed upon copanlisib exposure in the resistant cell lines, relative to the total-PI3K and total-AKT (Fig. 3B). Thereafter, these PI3K inhibitor resistant BCa cell lines were subjected to RNA-sequencing, along with their parental cell lines. Integrative analysis of transcriptome identified 167 and 610 mRNAs with significantly altered expression (fold change > 2, P adj < 0.05) in 5637-R cells and UM-UC-3 -R cells, respectively, (Fig. 3C). KEGG analysis showed that these differentially expressed genes were associated with JAK-STAT and ErbB signaling pathway activation in both 5637 and UM-UC-3 cells, with JAK-STAT exhibiting more marked enrichment (Fig. 3D). Besides, we also performed a human phospho-RTK antibody array encompassing 71 types of kinase receptors to elucidate potential pathways implicated in PI3K inhibitor resistance. Again, a more pronounced increase in tyrosine phosphorylation of JAK1 was detected in both 5637-R and UM-UC-3-R cell lines than in parental cells (Fig. 3E, F). The phosphorylation of STAT3, which is a classical downstream transcriptional factor of JAK1, was also significantly elevated in copanlisib resistant BCa cell lines (Fig. 3G). Consequently, these data reinforce the potential of JAK1-STAT3 signaling activation to drive PI3K inhibitor resistance in BCa cells.

### PI3K inhibition demonstrates synergistic anti-BCa effects with STAT3 inhibitors in vitro

**Fig. 4:**
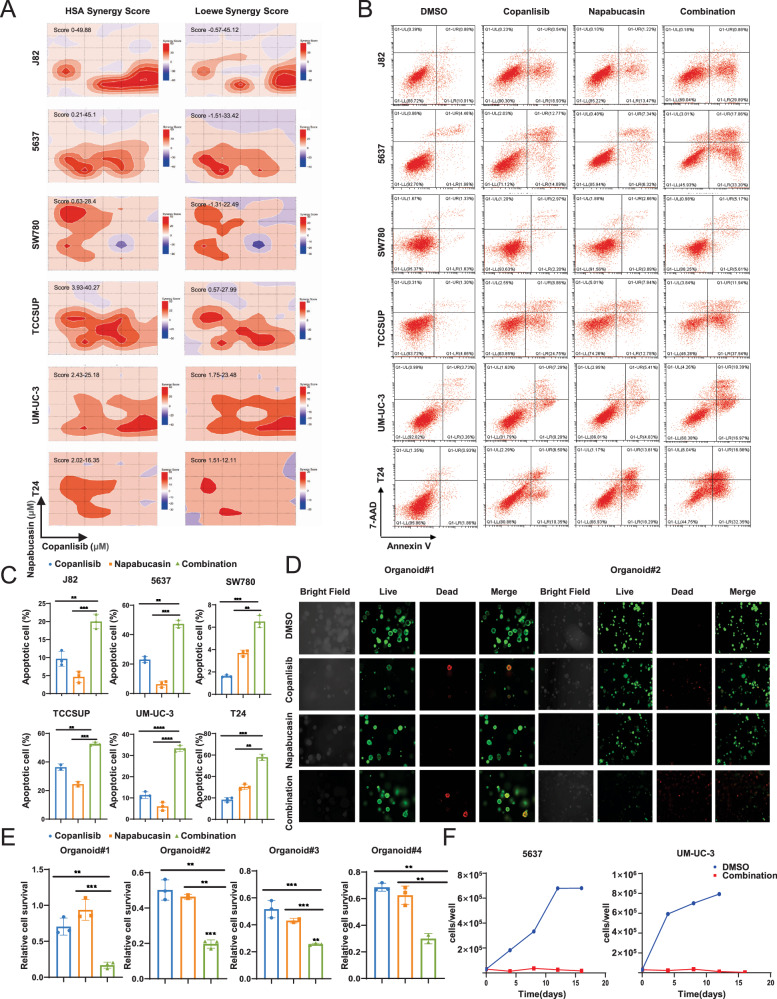
In vitro efficacy of combined PI3K and STAT3 inhibition. **A** The synergistic score between copanlisib and napabucasin was determined using Loewe and HSA synergy analysis in six BCa cells. Representative images (**B**) and quantification (**C**) of apoptosis assay in six BCa cells treated with DMSO, copanlisib, napabucasin or combination respectively. **D** Representative images of Live/Dead staining assay in organoid models treated with DMSO, copanlisib, napabucasin or combination respectively. **E** Organoids were treated with DMSO, copanlisib, napabucasin or combination in a CellTiter-Glo 3D cell viability assay. **F** Population doublings of 5637 and UM-UC-3 cells treated with DMSO or copanlisib and napabucasin for 20 days. Data are presented as mean ± SD of independent samples with individual data points shown; *P* values were assessed by two-tailed Student t test in comparison with single-agent cells group (**C**, **E**); *, *P* < 0.05; **, *P* < *0.01*; ***, P < *0.001*; ****, P < *0.0001*.

Next, we treated six BCa cell lines with copanlisib and napabucasin (STAT3 inhibitor), followed by HSA and Loewe model synergy analysis [27] to investigate whether inhibition of these two pathways can synergistically repress BCa cell survival. Our data showed a high synergy score for six BCa cells after a combined inhibition, which suggests a robust synergistic anti-BCa effect (Fig. 4A). In addition, we also observed synergistic suppression of cell viability in cells as indicated by a CI value less than 1, when cells were treated with PI3K and STAT3 inhibitors across a wide range of molar ratios (Supplementary Fig. S3B). Additionally, when the BCa cells were subjected to apoptosis assay by flow cytometry, our data showed that the apoptotic rate of cells treated with both drugs was significantly higher than that of cells treated with either drug alone (Fig. 4B, C). Moreover, we also treated our four BCa organoids with these two inhibitors and found that the combinatorial treatment with copanlisib and napabucasin induced organoid death to a greater extent than each single-drug treatment (Fig. 4D and Supplementary Fig. S3C), which is consistent with the result obtained from CellTiter-Glo 3D cell viability assay (Fig. 4E). Furthermore, we performed a long-term drug treatment of copanlisib and napabucasin in 5637 and UM-UC-3 cells for 20 days. Unlike the group treated with copanlisib alone, a durable cell growth inhibitory effect by the combinatory treatment lasted until the end of the experiment (Fig. 4F).

Taken together, our data indicate that the inhibition of STAT3 has a synergistic anti-BCa effect with PI3K inhibition and the combinatorial strategy holds promise for overcoming resistance to copanlisib.

### PI3K activates the JAK1-STAT3 signaling via downregulating PTPN11 expression

**Fig. 5:**
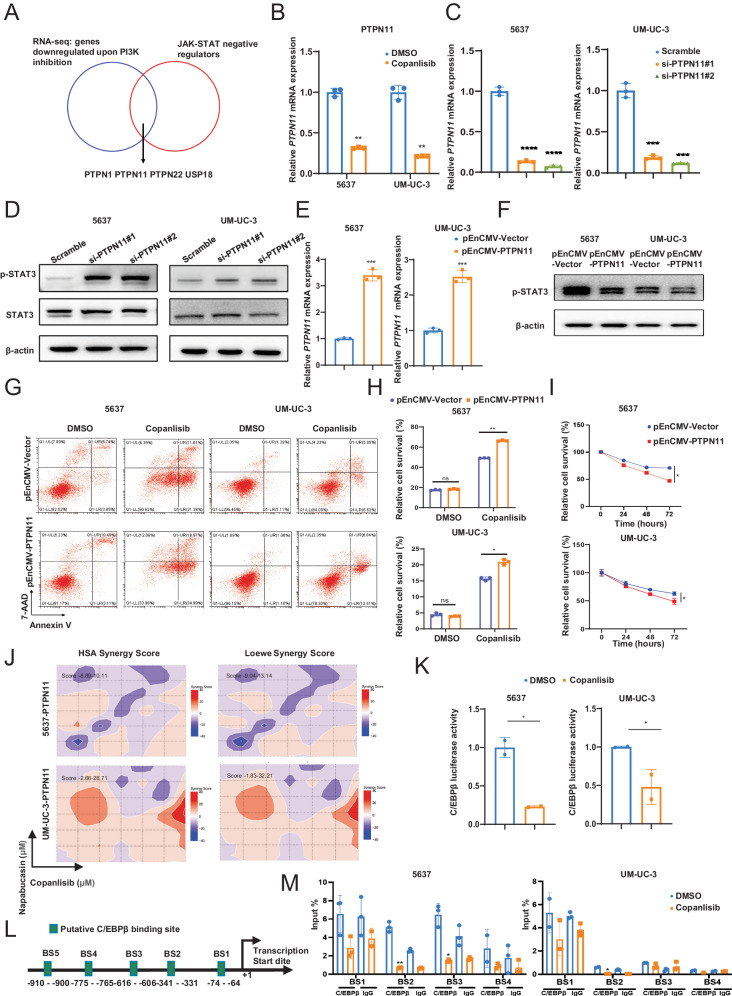
Feedback activation of JAK1-STAT3 signaling via downregulation of PTPN11 expression. **A** Venn diagram showed overlapping of the negative regulators of JAK-STAT pathway and down-regulated genes in resistant cells screened by RNA-seq. **B** The relative mRNA levels of PTPN11 in 5637 and UM-UC-3 cells with or without copanlisib treatment. **C** Knockdown of PTPN11 by two different siRNAs in 5637 and UM-UC-3 cells were verified by qPCR. **D** Western blot analysis of phosphorylated STAT3 and total STAT3 protein levels after transfection with indicated PTPN11 siRNAs in 5637 or UM-UC-3 cells. **E** Overexpression of PTPN11 by pEnCMV-PTPN11 plasmid in 5637 and UM-UC-3 cells were verified by qPCR. **F** Western blot analysis of phosphorylated STAT3 and total STAT3 protein levels after transfection with pEnCMV-PTPN11 plasmid in 5637 or UM-UC-3 cells. Representative images (**G**) and quantification (**H**) of apoptosis assay in the effect of PTPN11 overexpression in 5637 or UM-UC-3 cells on copanlisib sensitivity. **I** CCK8 assay explored the effect of PTPN11 overexpression in 5637 or UM-UC-3 cells on copanlisib sensitivity. **J** The synergistic score between copanlisib and napabucasin was determined using Loewe and HSA synergy analysis in cells overexpression of PTPN11. **K** Relative luciferase activity of C/EBPβ upon PI3K inhibition in 5637 or UM-UC-3 cells. Firefly luciferase activity was measured and normalized to Renilla luciferase activity. **L** Putative binding site of C/EBPβ in PTPN11 promoter region. **M** ChIP assay analyzed the recruitment of C/EBPβ at PTPN11 promoter in 5637 and UM-UC-3 cells treated with copanlisib. Data are presented as mean ± SD of independent samples with individual data points shown; *P* values were assessed by two-tailed Student t test (**B**, **C**, **E**, **H**, **L**, **M**) or equivalent ANCOVA (**I**) in comparison with the vehicle (DMSO) group (**B**, **H**, **L**) or the Scramble group (**C**, **E**, **M**); *, *P* < 0.05; **, *P* < *0.01*; ***, P < *0.001*; ****, P < *0.0001*.

As reported, the JAK-STAT pathway is directly regulated by negative regulatory factors, including suppressor of cytokine signaling (SOCS family), ubiquitin-specific peptidase 18 (USP18), IFN-stimulated gene 15 (ISG15), and multiple phosphatases [28], so we further explored whether certain negative regulatory factors affect the sensitivity of BCa cells to PI3K inhibitor by affecting JAK1-STAT3 signaling. We analyzed the down-regulated genes in 5637-R and UM-UC-3-R cells which were identified by RNA-seq, and four negative regulatory factors were screened out, including PTPN1, PTPN11, PTPN22 and USP18 (Fig. 5A).

To further ascertain the RNA-seq results, we validated the expression level of these four genes by qPCR. Among all the tested genes, PTPN1 and PTPN11 expression were significantly decreased in copanlisib-treated BCa cells, while USP18 or PTPN22 did not show such changes (Fig. 5B, Supplementary Fig. S4A, B). Furthermore, the expression of PI3KCA was found to be positively correlated with PTPN1 or PTPN11 expression, as indicated by the Gene Expression Profiling Interactive Analysis (GEPIA) database (http://gepia.cancer-pku.cn/, Supplementary Fig. S4C). Therefore, we focused our further investigations on PTPN1 and PTPN11.

To investigate the function of PTPN1 or PTPN11 in the JAK1-STAT3 pathway, we then knocked down the expression of PTPN1 or PTPN11 by siRNAs in BCa (5637 and UM-UC-3) cells, which in turn induced an increased level of phosphorylated STAT3 (Fig. 5C, D, Supplementary Fig. S4D–F). By contrast, STAT3 phosphorylation was attenuated upon overexpression of PTPN1 or PTPN11 (Fig. 5E, F, Supplementary Fig. S4G–I), implying that PTPN1 or PTPN11 deficiency leads to the activation of JAK1-STAT3 signaling. Functionally, cell apoptosis (Fig. 5G, H) and viability assay (Fig. 5I) showed that the introduction of PTPN11 increases the sensitivity of BCa cells to copanlisib. Moreover, synergistic growth inhibition was attenuated upon PTPN11-overexpression, as indicated by a low Loewe and HSA synergy score (Fig. 5J) and CI value exceeding 1 (Supplementary Fig. S3D), suggesting that PTPN11 contributes to PI3K inhibitor resistance. Conversely, overexpression of PTPN1 did not exert a significant effect on sensitivity to PI3K inhibitors (Supplementary Fig. S4J).

Next, we identified the transcription factors regulating the expression of PTPN11 after PI3K inhibition. It was previously reported that transcription factor C/EBPβ is regulated by the PI3K-AKT pathway. In line with the report, we found that C/EBPβ transcriptional activity was significantly suppressed upon treatment with the PI3K inhibitor by luciferase reporter assay (Fig. 5K). Besides, the analysis of the PTPN11 promoter region was performed using the PROMO(https://alggen.lsi.upc.es/cgibin/promo_v3/promo/promoinit.cgi?dirDB=TF_8.3) and JASPAR (https://jaspar.elixir.no/) bioinformatics databases. The analysis indicated five potential binding sites for C/EBPβ (BS1, BS2, BS3, BS4 and BS5, shown in Fig. 5L and Supplementary Fig. S4K). These sites were further tested by ChIP assays, which demonstrated that C/EBPβ primarily binds to BS2 (-341 to -331 bp) and BS3 (-616 to -606 bp) of the PTPN11 promoter. Importantly, direct binding of C/EBPβ to BS2 was suppressed after copanlisib treatment in both the 5637 and UM-UC-3 cell groups (Fig. 5M).

The above data leads us to conclude that inhibition of PI3K boosts the aberrant expression of PTPN11 and results in JAK1-STAT3 activation in BCa.

### The combination of PI3K and STAT3 inhibitors effectively suppresses BCa growth in vivo

**Fig. 6:**
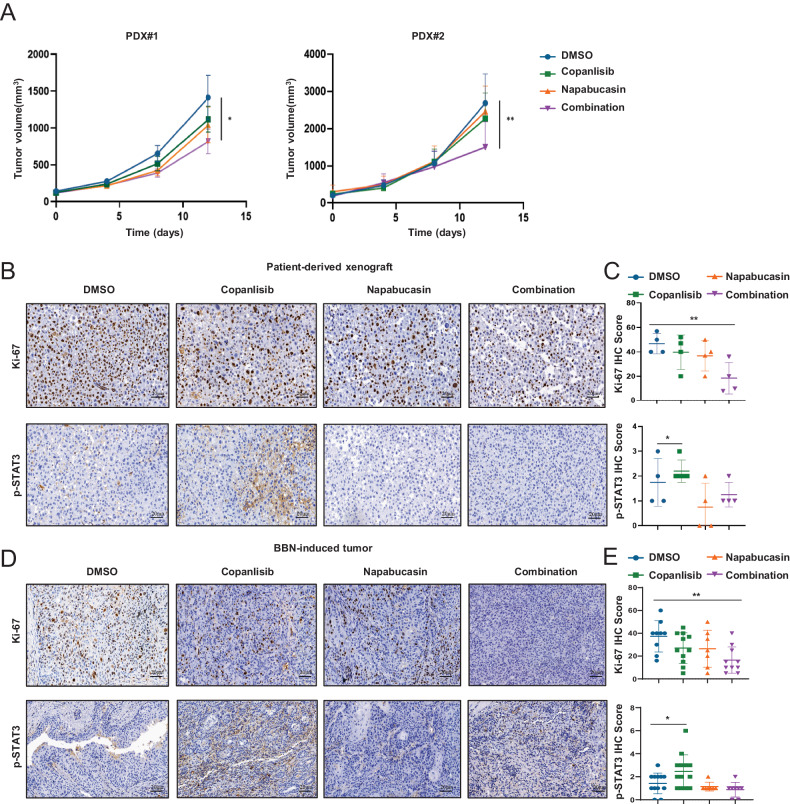
Combination PI3K and STAT3 inhibitor treatment is effective against BCa models in vivo. **A** Patient-derived xenograft tumors implanted into nude mice were randomized to DMSO, 10 mg/kg copanlisib, 4 mg/kg napabucasin, or the combination at the same doses in each cohort. Representative images (**B**) and quantification (**C**) of immunostaining for phosphorylated STAT3 and Ki-67 in Patient-derived xenograft was quantitatively measured three sections from every tumor tissue, Scale bar, 20 μm. Representative images (**D**) and quantification (**E**) of immunostaining for phosphorylated STAT3 and Ki-67 in BBN-induced tumor was quantitatively measured three sections from every tumor tissue, Scale bar, 20 μm. Data are presented as mean ± SD of independent samples with individual data points shown; *P* values were assessed by two-tailed Student t test in comparison with vehicle (DMSO) group (**C**, **E**, **F**); *, *P* < 0.05; **, *P* < *0.01*; ***, P < *0.001*; ****, P < *0.0001*.

We further verified the clinical relevance of our findings by evaluating the combined effects of PI3K and STAT3 inhibitors on BCa using primary human patient samples, tissues from bladder tumor bulks of two human patients (Supplementary Table S4) were isolated and injected subcutaneously into the flanks of immune-compromised mice (BALB/c-Nude) when the tumors reached 100 mm^3^, mice were randomly divided into four groups and subjected with DMSO, copanlisib, napabucasin, or combination (copanlisib and napabucasin) treatments. As expected, copanlisib in combination with napabucasin significantly retarded tumor growth compared with either agent alone (Fig. 6A). Additionally, we examined the levels of Ki67 and phosphorylated STAT3 expression by IHC and found that combinational treatment remarkably inhibited cell proliferation. Consistent with our in vitro results, copanlisib treatment also elevated the expression of phosphorylated STAT3 (Fig. 6B, C). Similar IHC staining results were also obtained in the BBN-induced BCa model (Fig. 6D, E). Collectively, our findings illustrate that the PI3K inhibitor copanlisib could enhance phosphorylated STAT3 levels, and the combination of PI3K and STAT3 significantly suppressed BCa growth in vivo.

## Discussion

In recent years, the application of targeted therapies has achieved rapid development in various types of cancer [29], notably in breast and lung cancer. In HER2-positive early breast cancer, patients treated with HER2 inhibitor had exciting survival rates which exceeded 90%; Similarly, in lung cancer, the ORR of EGFR inhibitor treatment in patients harboring EGFR mutation reaches 75% [30], all of which suggested that targeted therapies have a growing prominence in cancer patient management and drug development. Given its high tumor mutation burden, advanced BCa is likely to benefit from targeted therapy [31]. However, pan-FGFR tyrosine kinase inhibitor erdafitinib is the only FDA-approved targeted therapy for advanced BCa so far, with a limited response rate and a broad side effect profile [8, 32]. Thus, new strategies of targeted therapy for advanced BCa are necessarily and possibly to be developed.

In the current study, we discovered that PI3K is a promising therapeutic target of BCa, which is indicated by the HTS of small molecular inhibitors and aberrant PI3K activation in a broad panel of BCa cells. It is well-known that PI3K signaling plays an essential role in tumor development and survival in many cancers, and hyperactivation of the PI3K/AKT pathway leads to enhanced cell proliferation, migration, invasion and correlates with a worse prognosis [33, 34]. In BCa, PI3K signaling promotes cancer cell progression by driving epithelial-to-mesenchymal transition (EMT) and immune escape [35, 36]. PI3K inhibitors, which impair the activation of PI3K kinases, have shown promising therapeutic efficacy and have been clinically approved for use in several solid tumor. Evidence suggests that patients with relapsed or refractory indolent lymphoma treated with PI3K inhibitor exhibit an ORR of 59% [37], also significantly prolonged progression-free survival for patients harbor PIK3CA mutation in advanced breast cancer. Likewise, PI3K inhibitor revealed certain clinical activity in BCa therapy. In a phase II trial, seven of thirteen patients with metastatic urothelial carcinoma demonstrated a response to PI3K inhibitor buparlisib, with an ORR of 53.84% [20]. In line with this, our data also showed a potent anti-tumor impact of pan-PI3K tyrosine kinase inhibitor copanlisib in vivo and in vitro BCa models. Unfortunately, chronic exposure to copanlisib could lead to the acquisition of resistance, mirroring clinical observations of eventual disease progression after an initial response to several months of treatment.

To our knowledge, no PI3K inhibitor has been approved by FDA for BCa treatment. One of the reasons may be associated with drug toxicity including fatigue and hyperglycemia, which limits the administration of the adequate dose to inhibit the target [20]. Another important possibility is that bypass signaling pathway activation reduces dependence on PI3K signaling for tumor growth and proliferation [21, 38]. Thus, there is an urgent need to develop rational combinatorial treatment strategies and identify predictive biomarkers to expand the use of PI3K inhibitors in BCa patients and reverse acquired resistance.

In our prolonged in vitro culture experiments, we observed that resistance to PI3K inhibitor mainly originates from intrinsic changes of BCa cells rather than microenvironmental interventions. Additionally, the initial effectiveness followed by rapid resistance suggests an acquired phenotype as opposed to innate resistance To verify the mechanism involved in copanlisib resistance, we constructed drug-resistant cell lines through months of exposure in vitro and then performed RNA-seq and phospho-RTK Array, we found that feedback activation of JAK1-STAT3 signaling upon PI3K blockade facilitated cell survival and caused treatment failure. Inhibition of STAT3 potentiated responses to PI3K inhibitor in vitro and in vivo, durably suppressing tumor growth, increasing apoptosis, and causing marked tumor regression. It has been reported that STAT3 plays a crucial role in various types of cancers [39], activation of STAT3 leads to increased tumorigenic ability and the transition of cancer stem cells (CSCs) in cancer via enhancing the epithelial-mesenchymal transition (EMT) [40]. Consistent with these findings, our previous research demonstrates the essential role of STAT3 signaling in regulating the self-renewal ability of CSCs in BCa [41]. Moreover, the upregulation of STAT3 has been implicated as a mechanism of drug resistance in a range of oncogene-addicted cancers. For instance, in myeloid leukemia, STAT3 activation was potentially involved in TKI resistance and resulted in a worse prognosis [42].

Our research provides a rationale for combined targeting of the PI3K and STAT3 pathways in advanced BCa, which overcomes the acquired resistance and extends the use of PI3K inhibitors to BCa patients. However, the cause of STAT3 activation in BCa remains unknown. JAK-STAT pathway is reported to be regulated by numerous negative regulatory factors [28], in our previous work, we found that reactivation of SOCS3 (a negative regulator of the JAK/STAT pathway) by DNA demethylation will repress the STAT3 pathway, and improve the chemotherapy efficacy in BCa [41]. Our current work suggested a mechanism of adaptive resistance by which PI3K inhibition augments STAT3 feedback via the downregulation of PTPN11 expression. PTPN11 serves as a negative regulator for STAT3 signaling, which could decrease STAT3 activation by recruiting to pTyr759 of glycoprotein-130 (gp130) cytokine receptor where it dephosphorylates STAT3, or direct STAT3 dephosphorylation [43]. Evidence also shows that there is cross-talk between PTPN11 and PI3K [44]. Indeed, PTPN11 levels were down-regulated in copanlisib-treated BCa cells, and PTPN11-overexpressing cells proved more sensitive to copanlisib than the parental cell line in cell apoptosis and viability assays, other reports also showed that dysregulation of PTPN11 expression or activity causes many solid tumors, and also influence the sensitivity of cancer cells to anticancer drugs [45, 46]. Moreover, luciferase activity and ChIP assay validated that the activity of C/EBPβ was significantly suppressed by treating with copanlisib, which resulted in the transcription repression of PTPN11 and active JAK1-STAT3 pathway.

In conclusion, our study demonstrates the essential role of PI3K inhibitors in the targeted therapy of advanced BCa. Nonetheless, the rebound activation of JAK1-STAT3 signaling protects against cell death and maintains proliferation upon PI3K inhibition. Consequently, our results support using STAT3 inhibitor to potentiate PI3K inhibitor efficacy in the treatment of advanced BCa. Further clinical investigation that combines PI3K and STAT3 inhibitors in advanced BCa, and exploration of this combination in other solid tumors, should be considered in the future.

### Supplementary information

Supplementary Information

Supplementary Materials and Methods

Supplementary Table 1

Supplementary Table 2

Supplementary Table 8

## Data Availability

RNA-seq that support the findings of this study have been deposited in the NCBI database under the accession number PRJNA1124265. All other data supporting the findings of this study are available from the corresponding author on reasonable request. Source data are provided in this paper.
