# Supplementary material for: Combined Inhibition of PI3K and STAT3 signaling effectively inhibits bladder cancer growth
Source: Oncogenesis. 2024 Jul 27;13(1):29. doi: 10.1038/s41389-024-00529-y (PMC11283499; doi:10.1038/s41389-024-00529-y)
Supplement: Supplementary file 1 — Supplementary Information [file 41389_2024_529_MOESM1_ESM.pdf]

Supplemental Figures

A

	Enzastaurin	Copanlisib	Infigratinib	Everolimis	Ipatasertib	Ulixertinib	Sorafenib	Trametinib	QNZ	PNU 74654	Tofacitinib
SW780	15.70581	27.22275	24.90619	7.339549	9.625787	5.851174	10.28032	11.02279	0.302951	-1.20947	-6.88307
5637	11.89683	55	18.31241	9.197203	13.84159	7.456497	11.38006	-7.70059	8.75386	5.309877	-18.175
T24	19.71901	23.00953	12.195	14.85249	14.64597	8.728189	15.83093	2.328802	14.44553	8.917223	-7.81563
J82	17.05134	18.09133	1.49306	0.201885	0.409894	-2.92903	12.92604	-7.90506	5.404586	8.240846	-10.3451
UM-UC-3	13.11329	12.35333	17.72763	15.09709	22.22612	12.92083	18.72334	21.95774	26.28942	17.59612	-3.67615
TCCSUP	15.59399	35	32.19117	15.19081	21.51653	9.807786	13.96966	-9.89415	10.70324	4.384544	39.19667

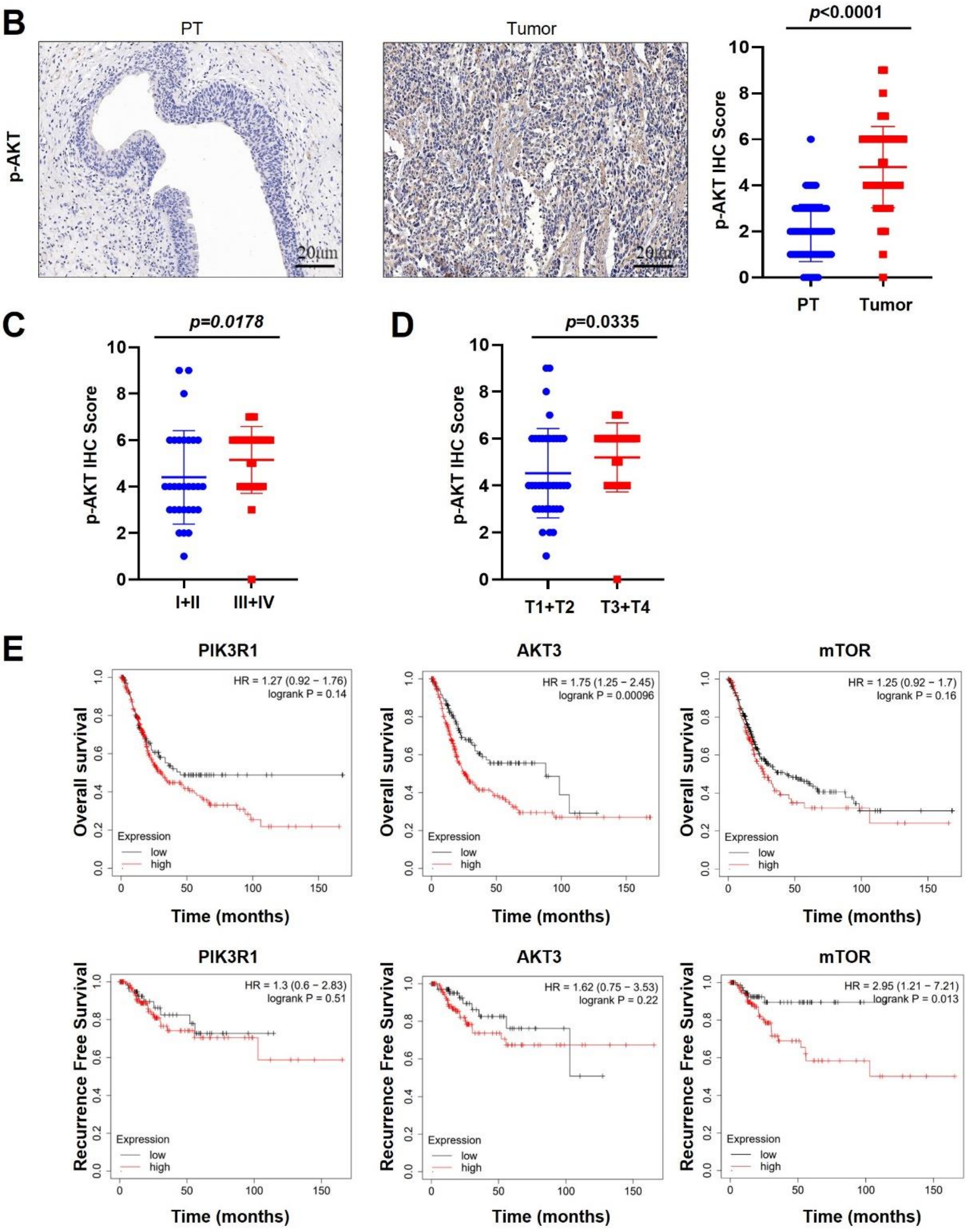

Supplementary Fig S1. PI3K signaling is activated in BCa. (A) The differences in inhibition rates of various small molecule inhibitors between tumor cells and normal cells. **(B)** Representative images of phosphorylated AKT IHC staining in clinical BCa samples (n =63) and IHC score of phosphorylated AKT in tumor and matched para-tumor tissues. PT, para-tumor tissues. Scale bars: (20 μm). **(C)** Phosphorylated AKT expression was analyzed based on the clinical stage. **(D)** Phosphorylated AKT expression was analyzed based on the tumor invasion. **(E)** PI3K pathway-related genes (PIK3R1, ATK3 and mTOR) are also associated with shorter overall survival and progression free survival. Data are presented as mean ± SD of independent samples with individual data points shown; P values were assessed by two-tailed Student t test in comparison with vehicle group **(B-D)**; *, $P < 0.05$; **, $P < 0.01$; ***, $P < 0.001$; ****, $P < 0.0001$.

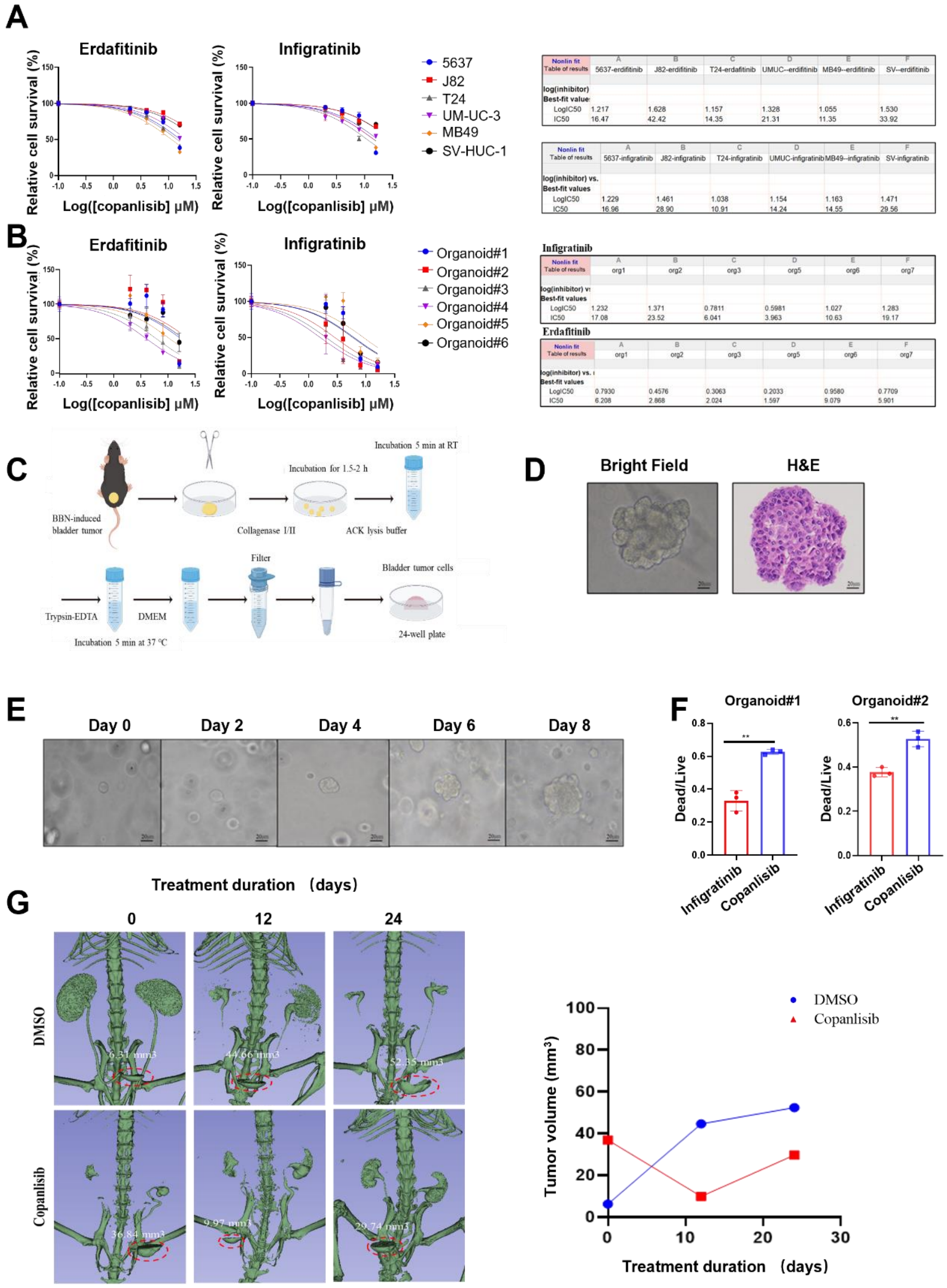

Supplementary Fig S2. The anti-tumor effect of PI3K inhibitor in BCa models. (A) IC_{50} assay of six bladder cancer cell lines with copanlisib or infigratinib treatment at indicated concentrations for 48 h. **(B)** IC_{50} assay of six organoids with copanlisib or infigratinib treatment at indicated concentrations for 48 h. **(C)** A schematic depicted the establishment

of mouse organoids from BBN-induced orthotopic tumor models. **(D)** The hematoxylin-eosin (H&E) staining of paraffin sections and bright-field images of the organoids, Scale bar, (20 μm). **(E)** Morphological changes of the organoids for a culture of 8 days. **(F)** Quantification of Live/Dead staining assay in organoid models treated with DMSO, infigratinib or copanlisib respectively. **(G)** Representative images and quantification of micro-computed tomography images from BBN-induced orthotopic tumor models treated with DMSO or copanlisib. Red dotted line circled the solid tumor. Quantitative values of each picture refers to tumor volume calculated by 3D Slicer, version 5.2.2. Data are presented as mean ± SD of independent samples with individual data points shown. Data are presented as mean ± SD of independent samples with individual data points shown; P values were assessed by two-tailed Student t test in comparison with vehicle (DMSO) group **(F, G)**; *, $P < 0.05$; **, $P < 0.01$; ***, $P < 0.001$; ****, $P < 0.0001$.

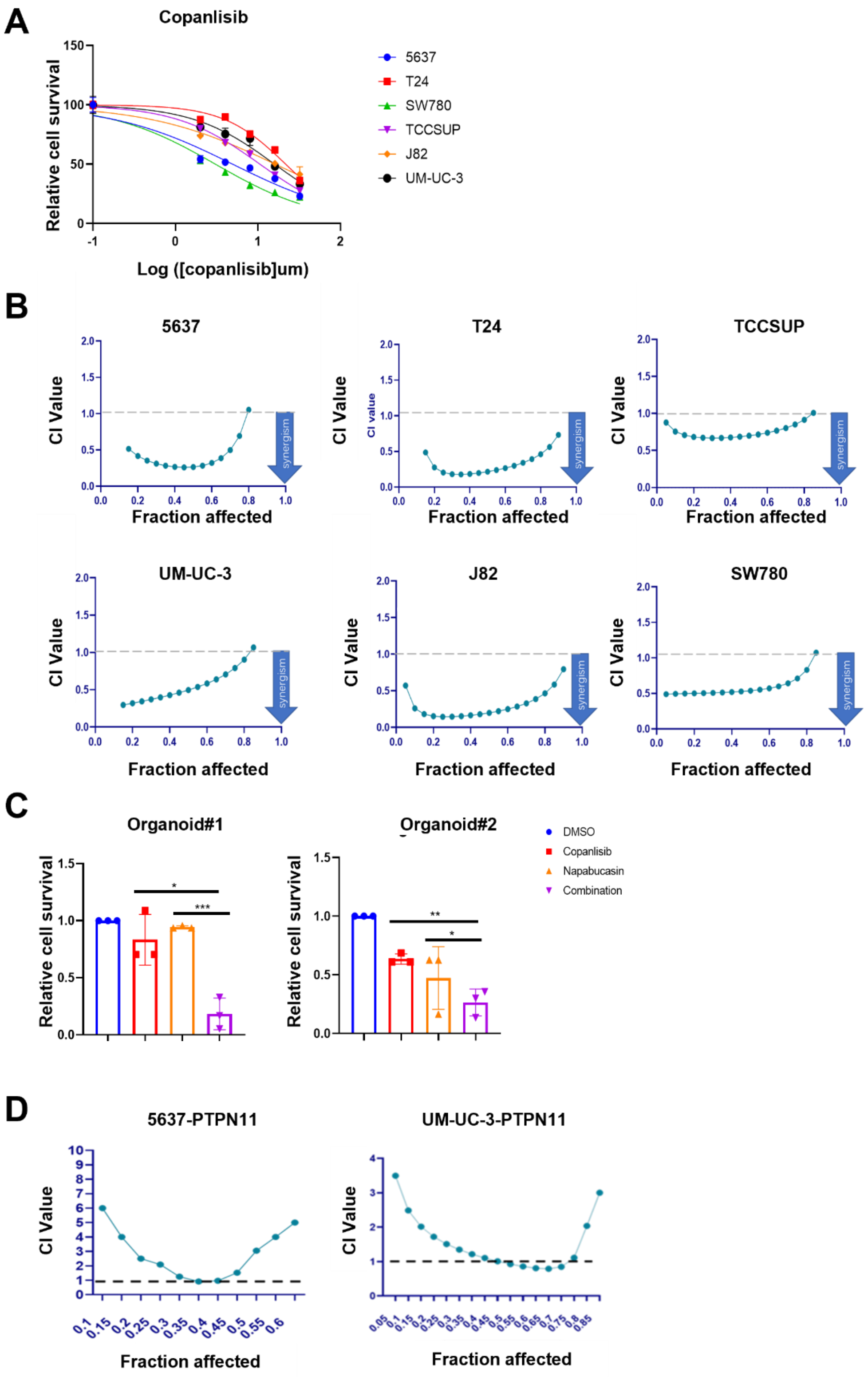

Supplementary Fig S3. Chou-Talalay analysis of BCa cells with/without PTPN11 overexpression. (A) The IC50 values of copanlisib in various BCa cell lines. **(B)** Chou-Talalay analysis of six BCa cells treated with varying concentrations of PI3K inhibitors

(copanlisib) and STAT3 inhibitors (napabucasin). Synergism was shown as CI < 1 at the optimal effect level (Fa > 0.75). The mean percentage of growth inhibition derived from n = 3 independent CCK8 assay experiments was used to calculate the CI value. **(C)** Quantification of Live/Dead staining assay in organoid models treated with DMSO, copanlisib, napabucasin or combination respectively. **(D)** Chou–Talalay analysis of PTPN11-overexpression 5637 and UM-UC-3 cells, cells were treated with varying concentrations of PI3K inhibitors (copanlisib) and STAT3 inhibitors (napabucasin). The mean percentage of growth inhibition derived from n = 3 independent CCK8 experiments was used to calculate the CI value. Data are presented as mean ± SD of independent samples with individual data points shown; P values were assessed by two-tailed Student t test in comparison with vehicle (DMSO) group **(C)**; *, *P < 0.05;* **, *P < 0.01*; ***, *P < 0.001*; ****, *P < 0.0001*.

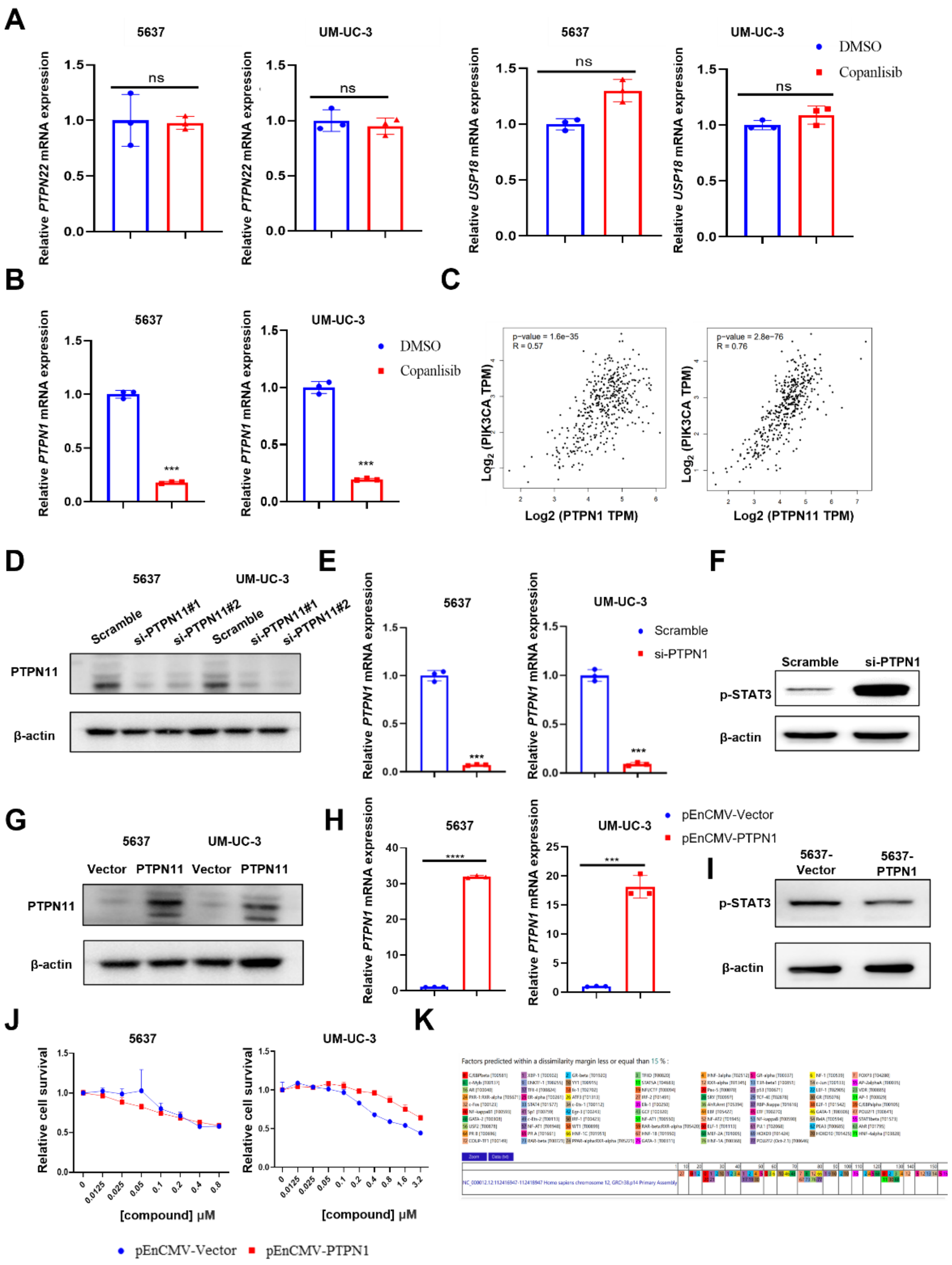

Supplementary Fig S4. Feedback activation of JAK1-STAT3 signaling via PTPN11.

(A) The relative mRNA levels of PTPN22 or USP18 in 5637 and UM-UC-3 cells with or without copanlisib treatment. **(B)** The relative mRNA levels of PTPN1 in 5637 and UM-UC-3 cells with or without copanlisib treatment. **(C)** Correlation between the expression level of PIK3CA and PTPN11 in BCa tumors based on GEPIA database. **(D)** Knockdown of PTPN11 by siRNAs in 5637 and UM-UC-3 cells were verified by Western blot. **(E)** Knockdown of PTPN1 by siRNAs in 5637 and UM-UC-3 cells were verified by qPCR. **(F)** Western blot analysis of phosphorylated STAT3 and total STAT3 protein levels after transfection with indicated PTPN11 siRNAs in 5637 or UM-UC-3 cells. **(G)** Overexpression of PTPN11 by pEnCMV-PTPN11 plasmid in 5637 and UM-UC-3 cells were verified by Western blot. **(H)** Overexpression of PTPN1 by pLVX-PTPN1 plasmid in 5637 and UM-UC-3 cells were verified by qPCR. **(I)** Western blot analysis of phosphorylated STAT3 after transfection with indicated PTPN1 plasmid in 5637 cells. **(J)** CCK8 assay explores the effect of PTPN1 overexpression in 5637 or UM-UC-3 cells on copanlisib sensitivity. **(K)** Prediction of transcription factor binding site in the promoter of PTPN11 based on PROMO version 8 database. Data are presented as mean ± SD of independent samples with individual data points shown; P values were assessed by two-tailed Student t test in comparison with vehicle group **(A-B, E, H)**; *, $P < 0.05$; **, $P < 0.01$; ***, $P < 0.001$; ****, $P < 0.0001$.

Supplemental Tables

Supplementary Table S1. The STR profiling and mycoplasma contamination report of cell lines.

Supplementary Table S2. Patient clinical characteristics. Included as a separate file.

Supplementary Table S3. The correlation between phosphorylated PI3K/AKT expression and the clinicopathological feature

Parameter	n	p-PI3K		P value	p-AKT		P value
		low	high		low	high	
Gender				0.1197			0.9818
Male	61	29	32		31	30	
Female	2	2	0		1	1	
Age				0.6820			0.3740
< 65	30	15	15		17	13	
≥65	33	16	17		15	18	
Tumor ststus				0.0267			0.0155
T1+T2	38	23	15		24	14	
T3+T4	25	8	17		8	17	
TNM stage				0.0016			0.0163
I+II	30	21	9		20	10	
III+IV	33	10	23		12	21	
Lymph node Metastasis				0.0314			0.5238
+	18	5	13		8	10	
-	45	26	19		24	21	

Supplementary Table S4. Patient characteristics used for PDX generating.

Case	PDX#1	PDX#2
Gender	Male	Male
Age	63	70
Stage	T3bN0M0	T3bN0M0
Grade	High grade muscle invasive urothelial carcinoma	High grade muscle invasive urothelial carcinoma

Supplementary Table S5. Sequence of siRNAs used for gene knockdown.

siRNA Names	Sequence
PTPN1#1	AUAGGUACAGAGACGUCAGUU
PTPN1#2	CCAAGAAACUCGAGAGAUC
PTPN11#1	GAAGAAUGGAGAUGUCAUU
PTPN11#2	GGAGAACGGUUUGAUUCUU

Supplementary Table S6. Primers used for quantitative PCR.

Primer Names	Sequence
USP18 -Forward	TCTGGAG GGCAGTATGAG
USP18 -Reverse	TGGTAGTTAGGATTTCCGTAG
PTPN1 -Forward	TGGGTGAAGGAAGAGACCCA
PTPN1- Reverse	CCCACGACCCGACTTCTAAC
PTPN11 -Forward	ACAGCCGAAAAGAGGGTCAA
PTPN11 - Reverse	TGGGCTTTGAATTGTTGCAC
PTPN22 -Forward	AGGCAGACAAAACCTATCCTACA
PTPN22 – Reverse	TGGGTGGCAATATAAGCCTTG
BS1- Forward	CCGCTCCCACCCCAAAGTAGTAT

BS1- Reverse	GAGAATCGCTTGAACCCGGGAG
BS2- Forward	TGCTTCAGACTCCCAAGCAGCTG
BS2- Reverse	TAATCCCAGCACTTTGGGAGGCC
BS3- Forward	TTAGGCAGTGTCTTGCCCTGTCG
BS3- Reverse	GCCTGGGCAAAAAGCAAGACCTC
BS4- Forward	CGCGCCCAGCCTACTATCTTTAT
BS4- Reverse	CCGCTGCCACATCGATTTCATTC

Supplementary Table S7. Antibodies used for western blot and immunostaining.

Protein target	Catalog Number	Host species	Company
PI3K	4257	Rabbit	CST
Phosphorylated PI3K	4228	Rabbit	CST
STAT3	9139	Mouse	CST
Phosphorylated STAT3	9145	Rabbit	CST
AKT	9272	Rabbit	CST
Phosphorylated AKT	4060	Rabbit	CST
Cleaved Caspase 3	9664	Rabbit	CST
Ki-67	PA5-114437	Rabbit	Thermo
β-actin	3382	Rabbit	CST
C/EBPβ	PA5-27244	Mouse	Thermo

Supplementary Table S8. The chemicals used for HTS. Included as a separate file.
