# Supplementary material for: Combined Inhibition of PI3K and STAT3 signaling effectively inhibits bladder cancer growth
Source: Oncogenesis. 2024 Jul 27;13(1):29. doi: 10.1038/s41389-024-00529-y (PMC11283499; doi:10.1038/s41389-024-00529-y)
Supplement: Supplementary file 2 — Supplementary Materials and Methods [file 41389_2024_529_MOESM2_ESM.pdf]

Combined Inhibition of PI3K and STAT3 Signaling Effectively Inhibits Bladder Cancer Growth

Weidong Peng[1,2*], Haojie Zhang[3*], Mingwei Yin[4], Dejie Kong[2], Liping Kang[2], Xinkun Teng[2], Jingjing Wang[2], Zhimin Chu[2], Yating Sun[2], Pengpeng Long[2], Chengying Cui[2], Bin Lyu[2], Jinzhi Zhang[2], Han Xiao[5], Mingqing Wu[2], Yongqiang Wang[6], Yang Li[1,2,7].

1. Department of Epidemiology and Health Statistics, School of Public Health，Anhui Medical University, Hefei, China.
2. Department of Genetics, School of Life Science, Anhui Medical University, Hefei, China.
3. Department of Urology, Huadong Hospital, Fudan University, Shanghai, China.
4. Department of Pathophysiology, School of Basic Medical Sciences, Anhui Medical University, Hefei, China.
5. Department of Pathology, The First Affiliated Hospital of Anhui Medical University, Hefei, China.
6. Department of Urology, South China Hospital, Medical School, Shenzhen University, Shenzhen, China.
7. Anhui Province Key Laboratory of Urological and Andrological Diseases Research and Medical Transformation, Hefei, China.

* The authors contribute equally to this work.

Address correspondence to: Yang Li, Department of Genetics, School of Life

Science, Anhui Medical University, Hefei, Anhui 230031, China; Tel: 86-551-65160327; Email: liyang@ahmu.edu.cn. Yongqiang Wang, Department of Urology, South China Hospital, Medical School, Shenzhen University, Shenzhen 518116, China; Tel: 86-18033411112; Email: wangyongqiang@szu.edu.cn. Mingqing Wu, Department of Genetics, School of Life Science, Anhui Medical University, Hefei, Anhui 230031, China; Tel: 86-18133629980; E-mail: wumingqing93@163.com.

Competing Interests statement: The authors declare that they have no conflict of interest.

Supplementary Materials and Methods

Cell viability assay and flow cytometry

BCa Cells were seeded at a density of 6000 cells per well on 96-well plates and incubated overnight. Compounds were added the next day over a 6-point concentration range, and then cells were incubated for 2 days, depending on the growth rate of individual cell lines. The viability of cells was then measured by the CCK8 assay (Topscience C0005). Half-maximal inhibitory concentration (IC_{50}) values were determined by GraphPad Prism 8 (San Diego, USA) using a 3-parameter dose–response model, IC_{50} values were calculated as previously described.[1] For flow cytometry analysis, BCa cells with DMSO or compounds treated were stained using the Annexin V/7-AAD apoptosis detection kits (BD 88-8102-72) according to the manufacturer's instructions. The dot plot of AnnexinV-PE versus 7-AAD was used for the assessment of apoptosis. Cells that were Annexin V-PE and 7-AAD–negative were considered viable. Cells that were Annexin V-PE–positive and 7-AAD–negative were considered early apoptotic. Cells that were Annexin V-PE– and 7-AAD–positive were considered late apoptotic or already dead.

Phospho-RTK Array

Whole-cell lysates were obtained from parental and copanlisib-resistant cancer cell lines after a 24-hour incubation and applied to a Human Phospho-RTK

Array Kit (Raybiotech AAH-PRTK-1-4)) following the manufacturer's instructions. In brief, (300 μg) of protein from each sample was applied to the nitrocellulose array membranes with capture and control antibodies spotted in duplicate. A pan-p-Tyr antibody conjugated to horseradish peroxidase was then used to detect phosphorylated tyrosines by chemiluminescence. Signal intensities on the membranes were quantified using the image analysis software AlphaEaseFC (Alpha Innotech).

siRNA transfection

5637 and UM-UC-3 cells were seeded at 60% to 80% confluency in 6-well plates and allowed to attach overnight. Lipofectamine RNAi MAX Transfection Reagent (Thermo #13778075) was used to perform siRNA transfection according to the manufacturer's instructions. Gene knockdown was detected by Western blot or RT- quantitative real-time (qPCR) analysis after transfection for 72 hours. siRNAs were purchased from General Biol. Target sequences for siRNAs are shown in **Supplementary Table S5**.

Chromatin immunoprecipitation assays

Chromatin immunoprecipitation (ChIP) assay was performed using a Simple ChIP Assay Kit (Abcam PA5-27244) according to the manufacturer's instructions. Briefly, bladder cancer cells were first cross-linked with 1% formaldehyde for 10 minutes. Then glycine was added to a final concentration of (0.125 M) and incubation was continued for 5 minutes. Afterward, cells were

collected and lysed. After sonication, one-eleventh of the lysate was kept as an input sample, while five-elevenths of the lysate were immunoprecipitated with control IgG or anti-C/EBPβ antibody. Immunocomplexes from each sample were eluted, followed by treatment with RNase A and proteinase K. Both the input sample and the immunoprecipitated sample were analyzed by real-time PCR. The recruitment of C/EBPβ to PTPN11 was calculated relative to the input using the following formula: %Input=2^ (CT^{Input} – CT^{IP}) ×5×100%. Four sets of primers designed to cover the putative binding sites (BS) are listed in **Supplementary Table S6** and antibodies used for ChIP assay are listed in **Supplementary Table S7.**

High-throughput screening

High throughput screening (HTS) was performed using an automated 384-well platform, 5637 cells were screened against a custom library of 150 compounds. One thousand cells were plated per well and treated with each library compound at the concentration of (4 μM), with DMSO treated as control. Compounds were screened in duplicate and the growth inhibition was assessed by CCK8 assays at 2 days post drug treatment. The chemicals used for HTS are summarized in **Supplementary Table S8**.

Luciferase assay

BCa cells were seeded in 6-well plates and co-transfected with (1μg) firefly

luciferase reporter plasmid C/EBPβ-Luc Reporter Plasmid（Genomeditech GM-021060）and (100 ng) Renilla luciferase reporter plasmid pRL-TK (Promega, catalog no. E2241). Both firefly and Renilla luciferase activities were assessed 48 hours post-transfection with the Dual-Luciferase Reporter Assay System (Promega, catalog no. E1910), and the luciferase activity was calculated by normalizing firefly luciferase activity for transfection efficiency against Renilla luciferase activity (Promega).

RNA Extraction and RNA Sequencing

RNA was extracted from parental and resistant cells by TRIzol (Invitrogen catalog no. 15596026). Total RNA was quantified and assessed for quality using a Nanodrop spectrophotometer (Thermo Fisher). Quantitative RT-PCR was performed as previously described [2]. The primers used for RT-PCR are listed in **Supplementary Table S6**.

RNA sequencing was performed and analyzed at GENEWIZ (genewiz.com, RRID: SCR_003177). Sequence reads were trimmed to remove possible adapter sequences and nucleotides with poor quality using Trimmomatic v.0.36. The trimmed reads were mapped to the Homo sapiens GRCh38 reference genome available on ENSEMBL using the STAR aligner v.2.5.2b. Differential expression analysis used the DESeq2 Bioconductor package in R.

Western blotting

Proteins extracted from BCa cells using RIPA buffer were loaded onto SDS–

polyacrylamide gels and then transferred to PVDF membranes. Membranes were blocked with 5% nonfat milk in TBS-T for 1 h at room temperature. Membranes were incubated overnight at 4°C with the primary antibodies. Antibodies are listed in **Supplementary Table S7**. After incubating the membranes with peroxidase-conjugated secondary antibodies (Cat. No. ZB-2301, ZSGB-BIO) for 2 h, the reaction was visualized by an enhanced chemiluminescence assay.

Immunohistochemistry

Paraffin sections of BCa tissue samples were antigen-retrieved, blocked, and processed as described previously [3]. Briefly, Formalin-fixed-paraffin-embedded samples sliced into (4 μm) sections were deparaffinized in xylene and graded alcohol, followed by hydration. Antigen retrieval was performed in a pressure cooker containing (0.01 M) sodium citrate buffer (pH 6.0) for 10 min. Tissue sections were incubated with either anti-Ki-67 (PA5-19462) or anti-phosphorylated PI3K (#4228) and phosphorylated STAT3 (#9145) overnight at 4°C. After blocking by endogenous peroxidase for 30 min, samples were incubated with the HRP-labeled sheep anti-mouse/rabbit IgG polymer (Cat. No. PV-6000, ZSGB-BIO) for 20 min at room temperature. Finally, 3,3′-Diaminobenzidine (DAB) was used as chromogen, and the sections were counterstained with hematoxylin. Antibodies are listed in **Supplementary Table S7**. The staining score was determined by the presence of yellow or brown-

yellow staining in the cell membrane or cytoplasm, indicating positive staining. For each section, ten high-magnification fields of view were randomly selected to count the positive expression in tumor tissues, excluding necrotic and mesenchymal areas.

Bioinformatic analyses

For the TCGA cohort, RNA sequencing data from 411 BCa samples and 19 noncancerous bladder samples were collected from the Genomic Data Commons data portal (https://portal.gdc.cancer.gov). For Gene Expression Omnibus (GEO) datasets, the two biggest bladder cancer cohorts, GSE13507 (n=223) and GSE31189 (n=92) were downloaded from the GEO (http://www.ncbi.nlm.nih.gov/geo/). GSEA and KEGG were conducted using the cluster Profiler R package. The code used for analysis has been uploaded to the Codeocean capsule. The Kaplan–Meier plotter offers a means of readily exploring the impact of a wide array of genes on patient survival in 21 types of cancer, with large sample sizes for the bladder carcinoma (n=405) cohorts. We therefore used this database to explore the association between PIK3CA expression and outcome in patients with BCa (https://kmplot.com/ analysis/).

Drug synergy analysis

For Combination index (CI) analysis, BCa cells were simultaneously exposed to indicated concentrations of PI3K and STAT3 inhibitors, and cell viability was

subsequently assessed using CCK8 assay. CI values were determined by the inhibition rate of the cells and calculated using CompuSyn software (https://www.combosyn.com/). CI values less than 1 indicated a synergistic interaction between compounds, while those equal to or more than 1.0 indicated additivity or antagonism. Multi-drug combination synergies were thoroughly examined and visually represented utilizing the R package synergyfinder (version 3.2.10), including two drug-drug interaction models: Highest Single Agent (HSA) and Loewe model models [4,5].

Tissue dissociation organoid culture

Tumor tissues from BBN-induced BCa models were washed twice with D-PBS (Gibco 14190144) and minced with scissors, then incubated in (10 mL) of the organoid culture media supplemented with Collagenase I (Sigma C0130), Collagenase II (Sigma C6885), Thermoprotease (Sigma T7902) at 37°C for 15 min. Dissociated tissues were spun down at (350 g) for 5 min, resuspended in (10 mL) of PBS, and spun down again. The tissues were resuspended in (5 mL) TrypLE recombinase (Gibco 12604-013) for 5 minutes. Next, dissociated tissues were resuspended with (10 mL) DMEM/F-12 (Gibco 12634010) and passed through a (100 μm) cell strainer (Corning 352360). Finally, cell clusters were spun down and resuspended in 60% Matrigel (Corning 356234) with 40% organoid culture media and plated in a (50 μL) drop in the middle of one well of a pre-coated 24-well plate. The drop was solidified by a 30-minute incubation

at 37°C and 5% CO_2. After solid drops formed, (600 μL) of the organoid culture media was added to the well, the medium was changed every 3-4 days.

Organoid viability assays

To plate organoids for analyses of drug response, organoids were collected 4-5 days after passaging and passed through a (100 μm) cell strainer (Corning no.352360) to eliminate large organoids. Subsequently, organoids were resuspended in 2% Matrigel /organoid mixed culture medium (15000-20000 organoids/mL) and then dispensed into ultralow-attachment 96-well plates (Corning 3474) in triplicate. Drug concentrations ranged from (0.1 μM) to (8 μM). Cell viability was assayed using CellTiter-Glo 3D (Promega G9683) according to the manufacturer's instructions following 2 days of drug incubation, and results were normalized to DMSO controls. Viability was also assessed using LIVE/DEAD staining (Thermo Fisher Scientific L3224) according to the manufacturer's instructions.

Reference

[1] Li Y, Deng H, Lv L, Zhang C, Qian L, Xiao J, et al. The mir-193a-3p-regulated ing5 gene activates the dna damage response pathway and inhibits multi-chemoresistance in bladder cancer. Oncotarget 2015;6(12):10195-206. 10.18632/oncotarget.3555.

[2] Gao Q, Zheng J, Ni Z, Sun P, Yang C, Cheng M, et al. The m(6)a methylation-regulated aff4 promotes self-renewal of bladder cancer stem cells. Stem Cells Int 2020;2020:8849218. 10.1155/2020/8849218.

[3] Peng W, Sheng Y, Xiao H, Ye Y, Kwantwi LB, Cheng L, et al. Lung adenocarcinoma cells promote self-migration and self-invasion by activating neutrophils to upregulate notch3 expression of cancer cells. Front Mol Biosci 2021;8:762729. 10.3389/fmolb.2021.762729.

[4] Jafari M, Mirzaie M, Bao J, Barneh F, Zheng S, Eriksson J, et al. Bipartite network models to design combination therapies in acute myeloid leukaemia. Nat Commun 2022;13(1):2128. 10.1038/s41467-022-29793-5.

[5] Ianevski A, He L, Aittokallio T, Tang J. Synergyfinder: a web application for analyzing drug combination dose-response matrix data. Bioinformatics 2020;36(8):2645. 10.1093/bioinformatics/btaa102.
