# Supplementary material for: Combined Inhibition of PI3K and STAT3 signaling effectively inhibits bladder cancer growth
Source: Oncogenesis. 2024 Jul 27;13(1):29. doi: 10.1038/s41389-024-00529-y (PMC11283499; doi:10.1038/s41389-024-00529-y)
Supplement: Supplementary file 3 — Supplementary Table 1 [file 41389_2024_529_MOESM3_ESM.pdf]

CBTCCCAS Certificate of STR Analysis

Report Date: 2024-03-18

1.Sample

SW 780 [SW-780, SW780]

2.Method and Procedure

Sample DNA was extracted by Microread Genomic DNA Kit.
PCR was amplified with STR Multi-amplification Kit(MicroreaderTM21 ID System).
PCR products were assayed with ABI 3730xl DNA Analyzer(Applied Biosystems®).
Data were analyzed using GeneMapperID-X software and then compared with the ATCC and DSMZdatabases for reference matching.

3.Results

D5S818	11, 12
D13S317	11, 12
D7S820	9, 10
D16S539	9, 11
vWA	16, 19
TH01	6
Amelogenin	X
TPOX	8
CSF1PO	10, 11

The above results were consistent with the DNA profiles reported by ATCC and DSMZ, and indicated no other human cell lines contamination.

Cell Bank,
Type Culture Collection,
Chinese Academy of Sciences
(CBTCCCAS)

中国科学院典型培养物保藏委员会
细胞库

Certificate of STR Analysis

1. **Sample**

   T24

2. **Methods**

   The sample DNA was analyzed in Genesky Biotechnologies. Inc., Shanghai.

   The PCR product was loaded into ABI 3730xl Genetic Analyzer and the STR profiling was generated by GeneMapper 5.0 software (Applied Biosystems®).

3. **Results**

Amelogenin:	X
CSF1PO:	10,12
D13S317:	12
D16S539:	9
D5S818:	10,12
D7S820:	10,11
THO1:	6
TPOX:	8,11
vWA:	17

The locations of the cell sample were consistent with the STR data of **T24** cell found in the databases of ATCC, DSMZ, JCRB and EXPASY.

There was no cross-contaminant of human source cell line.

Cell Bank/Stem Cell Bank
Center for Excellence in Molecular Cell Science
Chinese Academy of Sciences
Date: 2023/4/13

Certificate of STR Analysis

1. Sample

TCCSUP

2. Methods

The sample DNA was analyzed in Genesky Biotechnologies. Inc., Shanghai.

The PCR product was loaded into ABI 3730xl Genetic Analyzer and the STR profiling was generated by GeneMapper 5.0 software (Applied Biosystems®).

3. Results

Amelogenin:	X
CSF1PO:	10
D13S317:	11,14
D16S539:	9,11
D5S818:	12
D7S820:	8,9
THO1:	6,9.3
TPOX:	8
vWA:	14,16

The locations of the cell sample were consistent with the STR data of **TCCSUP** cell found in the databases of ATCC, DSMZ, JCRB and EXPASY.

There was no cross-contaminant of human source cell line.

Cell Bank/Stem Cell Bank

Center for Excellence in Molecular Cell Science

Chinese Academy of Sciences

Date: 2023/8/25

GENETIC TESTING BIOTECHNOLOGY

Cell Line Authentication Service
STR Profile Report

Sample Submitted By: Dr. Yang Li
Anhui Medical University

Email Address: liyang@ahmu.edu.cn
Sales Order: 240326L
Cell Line Designation: 5637
Date Sample Received: Mar 26th, 2024
Report Date: Mar 26th, 2024

Methodology: Twenty-one short tandem repeat (STR) loci plus the Amelogenin locus were amplified using the commercially available SiFaSTR™ 23 plex Kit. The cell line sample was processed using the ABI Prism® 3130 XL Genetic Analyzer. Data were analyzed using GeneMapper® ID v3.2 software (Applied Biosystems). Appropriate positive and negative controls were run and confirmed for each sample submitted.

Data Interpretation: Cell lines were authenticated using Short Tandem Repeat (STR) analysis as described in 2021 in ANSI Standard (ASN-0002) by the ATCC Standards Development Organization (SDO) and in Jamie L. Almeida et al., Authentication of Human and Mouse Cell Lines by Short Tandem Repeat (STR) DNA Genotype Analysis. Assay Guidance Manual. PMID: 23805434. Bookshelf ID: NBK144066.

GTB™ performs STR Profiling following ISO 9001:2008 and ISO/IEC 17025:2005 quality standards.
There are no warranties with respect to the services or results supplied, express or implied, including, without limitation, any implied warranty of merchantability or fitness for a particular purpose. Genetic Testing Biotechnology (GTB) is not liable for any damages or injuries resulting from receipt and/or improper, inappropriate, negligent or other wrongful use of the test results supplied, and/or from misidentification, misrepresentation, or lack of accuracy of those results. Your exclusive remedy against GTB and those supplying materials used in the services for any losses or damage of any kind whatsoever, whether in contract, tort, or otherwise, shall be, at GTB's option, refund of the fee paid for such service or repeat of the service.

NOTE: According to the recommendations of *IJC* on cell line authentication, the report is valid for 3 years since the issue date.

Technical Questions?
GTB Technical Support
+86-512-67486171
service@jsdna.org
Section 505, Yixin BLD
SIP, Suzhou, 215123
Jiangsu, P.R. China

Ordering Questions?
order@jsdna.org
GTB Corporation
+86-512-62806339
Section 303, Yixin BLD
SIP, Suzhou, 215123
Jiangsu, P.R. China

GENETIC TESTING BIOTECHNOLOGY

Cell Line Authentication Service
STR Profile Report

Sales Order: 240326L

	Test Results for Submitted Sample		ExPASy Reference Database Profile	
**Loci**	**Query Profile:** 5637		**Database Profile:** 5637	
Amelogenin	X			
D3S1358	15	17	15	17
D5S818	11	12	11	12
D2S1338	25			
TPOX	8		8	*9*
CSF1PO	11		11	
Penta D	11			
TH01	7	9	7	9
vWA	18		18	
D7S820	10	11	10	11
D21S11	36		36	
Penta E	10	12		
D10S1248	14			
D8S1179	10	16	10	16
D1S1656	15			
D18S51	16	18	16	18
D12S391	20			
D6S1043	16	20		
D19S433	13	15		
D16S539	9		9	
D13S317	11		11	
FGA	22		22	

The allele match algorithm compares the 13 core STR loci only, even though alleles from all loci will be reported when available.
Note: Loci highlighted in grey (13 core STR loci) can be made public to verify cell identity. In order to protect the identity of the donor, ***please do not publish*** *the allele calls from all the STR loci tested.*
The sample match is based on the reference data available at the time of comparison.

Explanation of Test Results

Cell lines with ≥80% match are derived from the same donor. Cell lines with between a 70% to 79% match require further profiling for authentication of relatedness. Cell lines with <70% match are very unlikely to be from the same donor.

- ☐ The submitted sample profile is human, but not a match for any profile in the ExPASy STR database.
- ☐ The submitted profile is an exact match for the following human cell line(s) in the ExPASy STR database (13 core STR loci):
- ☑ The submitted profile is similar to the following ExPASy human cell line(s): 5637 (97.44% match)

e-Si[illegible]nician: Digitally signed by Xuekun Chen
DN: cn=Xuekun Chen, o=Genetic Testing Biotechnology (Suzhou), ou=DNA Typing Section, email=order@jsdna.org, c=CN
Date: 2024.03.26 16:19:54 +08'00'

e-Signature, Reviewer: Digitally signed by Xiankun Zhao
DN: cn=Xiankun Zhao, o=Genetic Testing Biotechnology (Suzhou), ou=Supervision Section, email=service@jsdna.org, c=CN
Date: 2024.03.26 16:26:49 +08'00'

More information

Addendum: Electropherogram for the customer's sample set 1 of 1

GENETIC TESTING BIOTECHNOLOGY

Cell Line Authentication Service
STR Profile Report

Sample Submitted By: Dr. Yang Li
Anhui Medical University
Email Address: liyang@ahmu.edu.cn
Sales Order: 240326N
Cell Line Designation: J82
Date Sample Received: Mar 26th, 2024
Report Date: Mar 26th, 2024

Methodology: Twenty-one short tandem repeat (STR) loci plus the Amelogenin locus were amplified using the commercially available SiFaSTR™ 23 plex Kit. The cell line sample was processed using the ABI Prism® 3130 XL Genetic Analyzer. Data were analyzed using GeneMapper® ID v3.2 software (Applied Biosystems). Appropriate positive and negative controls were run and confirmed for each sample submitted.

Data Interpretation: Cell lines were authenticated using Short Tandem Repeat (STR) analysis as described in 2021 in ANSI Standard (ASN-0002) by the ATCC Standards Development Organization (SDO) and in Jamie L. Almeida et al., Authentication of Human and Mouse Cell Lines by Short Tandem Repeat (STR) DNA Genotype Analysis. Assay Guidance Manual. PMID: 23805434. Bookshelf ID: NBK144066.

GTB™ performs STR Profiling following ISO 9001:2008 and ISO/IEC 17025:2005 quality standards.
There are no warranties with respect to the services or results supplied, express or implied, including, without limitation, any implied warranty of merchantability or fitness for a particular purpose. Genetic Testing Biotechnology (GTB) is not liable for any damages or injuries resulting from receipt and/or improper, inappropriate, negligent or other wrongful use of the test results supplied, and/or from misidentification, misrepresentation, or lack of accuracy of those results. Your exclusive remedy against GTB and those supplying materials used in the services for any losses or damage of any kind whatsoever, whether in contract, tort, or otherwise, shall be, at GTB's option, refund of the fee paid for such service or repeat of the service.

NOTE: According to the recommendations of *IJC* on cell line authentication, the report is valid for 3 years since the issue date.

Technical Questions?
GTB Technical Support
+86-512-67486171
service@jsdna.org
Section 505, Yixin BLD
SIP, Suzhou, 215123
Jiangsu, P.R. China

Ordering Questions?
order@jsdna.org
GTB Corporation
+86-512-62806339
Section 303, Yixin BLD
SIP, Suzhou, 215123
Jiangsu, P.R. China

GENETIC TESTING BIOTECHNOLOGY

Cell Line Authentication Service
STR Profile Report

Sales Order: 240326N

	Test Results for Submitted Sample		ExPASy Reference Database Profile	
**Loci**	**Query Profile:** J82		**Database Profile:** J82	
Amelogenin	X	Y		
D3S1358	16	18	16	18
D5S818	12	13	12	13
D2S1338	19			
TPOX	11	12	11	12
CSF1PO	10	11	10	11
Penta D	9			
TH01	9.3		9.3	
vWA	17	18	17	18
D7S820	9	11	9	11
D21S11	30		30	
Penta E	12	15		
D10S1248	13			
D8S1179	8	13	8	13
D1S1656	14			
D18S51	*10*	12	12	
D12S391	24			
D6S1043	19			
D19S433	12	13		
D16S539	11	12	11	12
D13S317	10	12	10	12
FGA	20	24	20	24

The allele match algorithm compares the 13 core STR loci only, even though alleles from all loci will be reported when available.
Note: Loci highlighted in grey (13 core STR loci) can be made public to verify cell identity. In order to protect the identity of the donor, ***please do not publish*** *the allele calls from all the STR loci tested.*
The sample match is based on the reference data available at the time of comparison.

Explanation of Test Results

Cell lines with ≥80% match are derived from the same donor. Cell lines with between a 70% to 79% match require further profiling for authentication of relatedness. Cell lines with <70% match are very unlikely to be from the same donor.

- ☐ The submitted sample profile is human, but not a match for any profile in the ExPASy STR database.
- ☐ The submitted profile is an exact match for the following human cell line(s) in the ExPASy STR database (13 core STR loci):
- ☑ The submitted profile is similar to the following ExPASy human cell line(s): J82 (97.87% match)

e-Signature, Technician: Digitally signed by Xuekun Chen
DN: cn=Xuekun Chen, o=Genetic Testing Biotechnology (Suzhou), ou=DNA Typing Section, email=order@jsdna.org, c=CN
Date: 2024.03.26 16:26:21 +08'00'

e-Signature, Reviewer: Digitally signed by Xiankun Zhao
DN: cn=Xiankun Zhao, o=Genetic Testing Biotechnology (Suzhou), ou=Supervision Section, email=service@jsdna.org, c=CN
Date: 2024.03.26 16:27:33 +08'00'

GUARANTEE CELL LINE AUTHENTICATION

More information

Addendum: Electropherogram for the customer's sample set 1 of 1

Cell Line Authentication Service
STR Profile Report

**Sample Submitted By:**	Dr. Yang Li<br>Anhui Medical University
**Email Address:**	liyang@ahmu.edu.cn
**Sales Order:**	240326K
**Cell Line Designation:**	SV-HUC-1
**Date Sample Received:**	Mar 26th, 2024
**Report Date:**	Mar 26th, 2024

Methodology: Twenty-one short tandem repeat (STR) loci plus the Amelogenin locus were amplified using the commercially available SiFaSTR™ 23 plex Kit. The cell line sample was processed using the ABI Prism® 3130 XL Genetic Analyzer. Data were analyzed using GeneMapper® ID v3.2 software (Applied Biosystems). Appropriate positive and negative controls were run and confirmed for each sample submitted.

Data Interpretation: Cell lines were authenticated using Short Tandem Repeat (STR) analysis as described in 2012 in ANSI Standard (ASN-0002) by the ATCC Standards Development Organization (SDO) and in Capes-Davis et al., Match criteria for human cell line authentication: Where do we draw the line? Int J Cancer. 2013;132(11):2510-9.

GTB™ performs STR Profiling following ISO 9001:2008 and ISO/IEC 17025:2005 quality standards.

There are no warranties with respect to the services or results supplied, express or implied, including, without limitation, any implied warranty of merchantability or fitness for a particular purpose. Genetic Testing Biotechnology (GTB) is not liable for any damages or injuries resulting from receipt and/or improper, inappropriate, negligent or other wrongful use of the test results supplied, and/or from misidentification, misrepresentation, or lack of accuracy of those results. Your exclusive remedy against GTB and those supplying materials used in the services for any losses or damage of any kind whatsoever, whether in contract, tort, or otherwise, shall be, at GTB's option, refund of the fee paid for such service or repeat of the service.

NOTE: According to the recommendations of *IJC* on cell line authentication, the report is valid for 3 years since the issue date.

Technical Questions?
GTB Technical Support
+86-512-67486171
service@jsdna.org
Section 505, Yixin BLD
SIP, Suzhou, 215123
Jiangsu, P.R. China

Ordering Questions?
order@jsdna.org
GTB Corporation
+86-512-62806339
Section 303, Yixin BLD
SIP, Suzhou, 215123
Jiangsu, P.R. China

GENETIC TESTING BIOTECHNOLOGY

Cell Line Authentication Service
STR Profile Report

Sales Order: 240326K

Loci	Test Results for Submitted Sample<br>**Query Profile:** SV-HUC-1		ExPASy Reference Database Profile<br>**Database Profile:** SV-HUC-1	
Amelogenin	X	Y	X	Y
D3S1358	14	17		
D5S818	12	14	12	14
D2S1338	17			
TPOX	8	10	8	10
CSF1PO	10	11	10	11
Penta D	13			
TH01	9	9.3	9	9.3
vWA	14		14	
D7S820	10	11	10	11
D21S11	29	30		
Penta E	10	20		
D10S1248	13	14		
D8S1179	14	16		
D1S1656	11	15		
D18S51	15	16		
D12S391	18			
D6S1043	11			
D19S433	14			
D16S539	11		11	
D13S317	12		12	*13*
FGA	21	22		

The allele match algorithm compares the 8 core loci plus amelogenin only, even though alleles from all loci will be reported when available.

*Note: Loci highlighted in grey (8 core STR loci plus Amelogenin) can be made public to verify cell identity. In order to protect the identity of the donor, **please do not publish** the allele calls from all the STR loci tested.*

The sample match is based on the reference data available at the time of comparison.

Explanation of Test Results

Cell lines with ≥80% match are considered to be related; i.e., derived from a common ancestry. Cell lines with between a 55% to 80% match require further profiling for authentication of relatedness.

- ☐ The submitted sample profile is human, but not a match for any profile in the ExPASy STR database.
- ☐ The submitted profile is an exact match for the following human cell line(s) in the ExPASy STR database (8 core loci plus Amelogenin):
- ☑ The submitted profile is similar to the following ExPASy human cell line(s): SV-HUC-1 (96.77% match)

e-Signature Technician: Digitally signed by Xuekun Chen
DN: cn=Xuekun Chen, o=Genetic Testing Biotechnology (Suzhou), ou=DNA Typing Section, email=order@jsdna.org, c=CN
Date: 2024.03.26 16:16:46 +08'00'

e-Signature Reviewer: Digitally signed by Xiankun Zhao
DN: cn=Xiankun Zhao, o=Genetic Testing Biotechnology (Suzhou), ou=Supervision Section, email=service@jsdna.org, c=CN
Date: 2024.03.26 16:27:53 +08'00'

GUARANTEE
CELL LINE AUTHENTICATION

More information

Addendum: Electropherogram for the customer's sample set 1 of 1

GENETIC TESTING BIOTECHNOLOGY

Cell Line Authentication Service
STR Profile Report

Sample Submitted By: Dr. Yang Li
Anhui Medical University

Email Address: liyang@ahmu.edu.cn
Sales Order: 240326M
Cell Line Designation: UM-UC-3
Date Sample Received: Mar 26th, 2024
Report Date: Mar 26th, 2024

Methodology: Twenty-one short tandem repeat (STR) loci plus the Amelogenin were amplified using the commercially available SiFaSTR™ 2[illegible] Kit. The cell line sample was processed using the ABI Prism® 31[illegible] Genetic Analyzer. Data were analyzed using GeneMapper® II[illegible] software (Applied Biosystems). Appropriate positive and ne[illegible] controls were run and confirmed for each sample submitted.

Data Interpretation: Cell lines were authenticated using Short Tandem Repeat [illegible] analysis as described in 2021 in ANSI Standard (ASN-0002) b[illegible] ATCC Standards Development Organization (SDO) and in Jar[illegible] Almeida et al., Authentication of Human and Mouse Cell Lin[illegible] Short Tandem Repeat (STR) DNA Genotype Analysis. Assay Gui[illegible] Manual. PMID: 23805434. Bookshelf ID: NBK144066.

GTB™ performs STR Profiling following ISO 9001:2008 and ISO/IEC 17025:2005 quality standar[illegible]
There are no warranties with respect to the services or results supplied, express or i[illegible] including, without limitation, any implied warranty of merchantability or fitness for a pa[illegible] purpose. Genetic Testing Biotechnology (GTB) is not liable for any damages or injuries resulti[illegible] receipt and/or improper, inappropriate, negligent or other wrongful use of the test results su[illegible] and/or from misidentification, misrepresentation, or lack of accuracy of those results[illegible] exclusive remedy against GTB and those supplying materials used in the services for any lo[illegible] damage of any kind whatsoever, whether in contract, tort, or otherwise, shall be, at GTB's [illegible] refund of the fee paid for such service or repeat of the service.

NOTE: According to the recommendations of *IJC* on cell line authentication, the report i[illegible] for 3 years since the issue date.

Technical Questions?
GTB Technical Support
+86-512-67486171
service@jsdna.org
Section 505, Yixin BLD
SIP, Suzhou, 215123
Jiangsu, P.R. China

Ordering Question[illegible]
order@jsdna.org
GTB Corporation
+86-512-62806339
Section 303, Yixin [illegible]
SIP, Suzhou, 2151[illegible]
Jiangsu, P.R. China

GENETIC TESTING BIOTECHNOLOGY

Cell Line Authentication Service
STR Profile Report

Sales Order: 240326M

	Test Results for Submitted Sample		ExPASy Reference Database Profile	
**Loci**	**Query Profile:** UM-UC-3		**Database Profile:** UM-UC-3	
Amelogenin	X			
D3S1358	17	18	17	18
D5S818	12		12	
D2S1338	23			
TPOX	10		10	
CSF1PO	10	11	10	11
Penta D	13			
TH01	6	9	6	9
vWA	17		17	
D7S820	8	9	8	9
D21S11	31		31	
Penta E	12			
D10S1248	14	15		
D8S1179	13		13	
D1S1656	15	17.3		
D18S51	14		14	
D12S391	22			
D6S1043	11	13		
D19S433	14	15		
D16S539	8	9	8	9
D13S317	8		8	
FGA	20	21	20	21

The allele match algorithm compares the 13 core STR loci only, even though alleles from all loci will be reported when available.
Note: Loci highlighted in grey (13 core STR loci) can be made public to verify cell identity. In order to protect the identity of the donor, ***please do not publish*** *the allele calls from all the STR loci tested.*
The sample match is based on the reference data available at the time of comparison.

Explanation of Test Results

Cell lines with ≥80% match are derived from the same donor. Cell lines with between a 70% to 79% match require further profiling for authentication of relatedness. Cell lines with <70% match are very unlikely to be from the same donor.

- ☐ The submitted sample profile is human, but not a match for any profile in the ExPASy STR database.
- ☑ The submitted profile is an exact match for the following human cell line(s) in the ExPASy STR database (13 core STR loci): UM-UC-3
- ☐ The submitted profile is similar to the following ExPASy human cell line(s):

e-Si[illegible]hnician: Digitally signed by Xuekun Chen
DN: cn=Xuekun Chen, o=Genetic Testing Biotechnology (Suzhou), ou=DNA Typing Section, email=order@jsdna.org, c=CN
Date: 2024.03.26 16:23:25 +08'00'

e-Signature, Reviewer: Digitally signed by Xiankun Zhao
DN: cn=Xiankun Zhao, o=Genetic Testing Biotechnology (Suzhou), ou=Supervision Section, email=service@jsdna.org, c=CN
Date: 2024.03.26 16:28:13 +08'00'

CELL LINE AUTHENTICATION

More information

Addendum: Electropherogram for the customer's sample set 1 of 1

支原体检测报告

编号	名称	支原体 PCR 结果	支原体测序结果	结论
1	T24<br>LOT20210531 P+3	阴性	/	合格
W	Negative（Water）	阴性	/	成立
P	Positive	阳性	/	成立

Marker	支原体
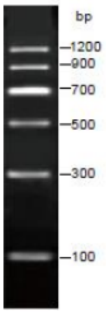	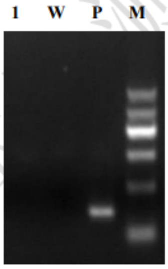

检测人：龙慧玲	审核人：龙慧玲

国家模式与特色实验细胞资源库

2021 年 05 月 31 日

中国科学院典型培养物保藏委员会 细胞库

细胞支原体检测报告

支原体检测报告

检测时间	编号	名称	批次	支原体 PCR 结果	结论
2018/09/13	1	TCCSUP	20180907	阴性	合格
	N	Negative		阴性	成立
	P	Positive		阳性	成立

Marker	支原体
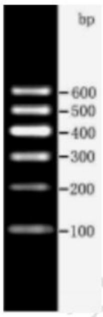	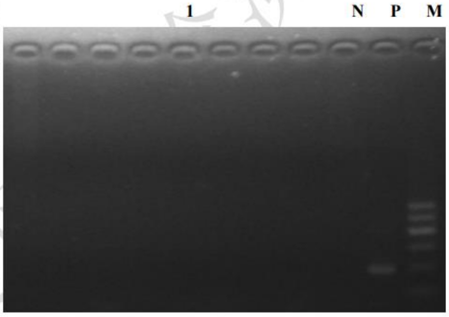

检测人	刘毅

国家模式与特色实验细胞资源库

细胞支原体检测报告

支原体检测报告

编号	名称	支原体 PCR 结果	支原体测序结果	结论
1	SW780	阴性	/	合格
W	Negative（Water）	阴性	/	成立
P	Positive	阳性	/	成立

Marker	支原体
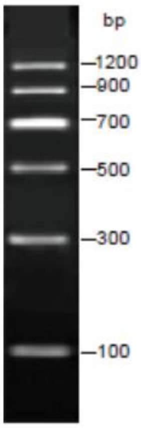	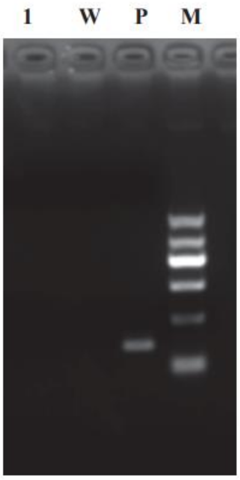

中国科学院典型培养物保藏委员会细胞库

2024/3/18

中国科学院典型培养物保藏委员会
细胞库

备注：

使用 TAKARA PCR Mycoplasma Detection Set(货号 6601) 支原体检测试剂盒检测，检测结果仅对送检样品有效。

中乔新舟 CELL RESEARCH

中乔新舟　Web:www.zqxzbio.com

支原体检测报告

支原体检测反应体系：

Myco-PCR-Mix	18ul
待检测细胞上清液	2ul

按照以上反应比例，在 PCR 管中将待检测样品上清液 2ul 加入到 18ul Myco-PCR-Mix 中，每次检测请用Positive control 模版作为阳性对照，用Negative control 模版作为阴性对照。PCR 反应程序为：95°C 5min，（95°C 30s，55°C 45s，72°C 45s）36 个循环，72°C 5min，10°C 保存。PCR 产物跑 1.5 % 琼脂胶，用DNA Marker 显示片段大小。待检测样品中如果出现与阳性对照大小一致（250 bp）的条带，说明样品被支原体感染，若无对应条带则说明无支原体污染。

跑胶结果如下图所示：

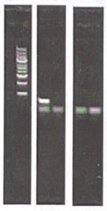

M 孔道为 DNA Marker

N 孔道为 Negative control

P 孔道为 Positive control

中乔新舟 CELL RESEARCH

中乔新舟　Web:www.zqxzbio.com

1 号孔道为待测 J82 细胞样品

2 号孔道为待测 J82 细胞样品复孔

3 细胞结论：由电泳图可知检测的 J82 细胞样品没有支原体污染。

（注：图谱中样品点样孔最下方的条带是引物二聚体，是PCR 检测时的常规现象，其亮暗程度通常是与阳性条带的亮度成反比的。PCR 的检测方法非常灵敏，一般极弱的阳性也可以看到有区别于二聚体的条带产生，结果可靠。）

上海中乔新舟生物科技有限公司 质检专用章

2022.03.10

检测人签名：金竹

5637 (human bladder cancer cell line)
Mycoplasma Detection Report

1. Reaction System

Myco-PCR-Mix	9 μl
Cell culture supernatant to be tested	1 μl

2. Test Procedure

According to the above reaction system, add 1 μl of the supernatant of the sample to be tested to 9 μl Myco-PCR-Mix in a PCR tube, use the Positive control template as the positive control, and use the Negative control template as the negative control. The PCR reaction program is: 95℃ 5min, (95℃ 15s, 56℃ 15s, 72℃ 5s), 35 cycles, 72℃ 5min, and store at 4℃. The PCR product is subjected to 1% agarose gel electrophoresis, and the molecular weight of the band is displayed with a DNA Marker. If a band with the same molecular weight as the positive control (about 500bp) appears in the sample to be tested, it means that the sample is contaminated with mycoplasma. If there is no corresponding band, it means that the sample is not contaminated with mycoplasma.

The electrophoresis image are shown in the figure below:

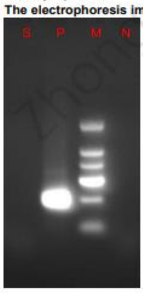

Lane S is 5637 (human bladder cancer cell line)
Lane P is Positive Control
Lane M is DNAMarker
Lane N is Negative Control

(Note: The band at the bottom of the sample loading wells in the gel image is the primer dimer, which is a common phenomenon in PCR detection. Its intensity is usually inversely proportional to the brightness of the positive band. The PCR detection method is very sensitive, and even a very weak positivity can be distinguished from the dimer, making the results reliable.)

3. Test result

Sample Name	Test Result	Test Criteria	Test Interpretation
5637 (human bladder cancer cell line)	-	Compare the positive control to see if there is characteristic band	Negative, no mycoplasma contamination

Shanghai Zhong Qiao Xin Zhou Biotechnology Co.,Ltd.
Reporter: Ke Li
Date: 2024-05-29

质检专用章

UM-UC-3

Mycoplasma Detection Report

1. Reaction System

Myco-PCR-Mix	9 µl
Cell culture supernatant to be tested	1 µl

2. Test Procedure

According to the above reaction system, add 1 µl of the supernatant of the sample to be tested to 9 µl Myco-PCR-Mix in a PCR tube, use the Positive control template as the positive control, and use the Negative control template as the negative control. The PCR reaction program is: 95℃ 5min, (95℃ 15s, 56℃ 15s, 72℃ 5s), 35 cycles, 72℃ 5min, and store at 4℃. The PCR product is subjected to 1% agarose gel electrophoresis, and the molecular weight of the band is displayed with a DNA Marker. If a band with the same molecular weight as the positive control (about 500bp) appears in the sample to be tested, it means that the sample is contaminated with mycoplasma. If there is no corresponding band, it means that the sample is not contaminated with mycoplasma.

The electrophoresis image are shown in the figure below:

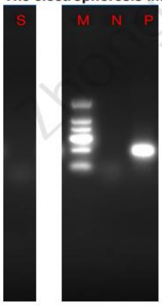

Lane S is UM-UC-3
Lane P is Positive Control
Lane M is DNAMarker
Lane N is Negative Control

(Note: The band at the bottom of the sample loading wells in the gel image is the primer dimer, which is a common phenomenon in PCR detection. Its intensity is usually inversely proportional to the brightness of the positive band. The PCR detection method is very sensitive, and even a very weak positivity can be distinguished from the dimer, making the results reliable.)

3. Test result

Sample Name	Test Result	Test Criteria	Test Interpretation
UM-UC-3	-	Compare the positive control to see if there is characteristic band	Negative,no mycoplasma contamination

Shanghai Zhong Qiao Xin Zhou Biotechnology Co.,Ltd.
Reporter：Ke Li
Date：2024-06-03

上海中乔新舟生物科技有限公司
质检专用章
Zhong Qiao Xin Zhou

SV-HUC-1

Mycoplasma Detection Report

1. Reaction System

Myco-PCR-Mix	9 µl
Cell culture supernatant to be tested	1 µl

2. Test Procedure

According to the above reaction system, add 1 µl of the supernatant of the sample to be tested to 9 µl Myco-PCR-Mix in a PCR tube, use the Positive control template as the positive control, and use the Negative control template as the negative control. The PCR reaction program is: 95℃ 5min, (95℃ 15s, 56℃ 15s, 72℃ 5s), 35 cycles, 72℃ 5min, and store at 4℃. The PCR product is subjected to 1% agarose gel electrophoresis, and the molecular weight of the band is displayed with a DNA Marker. If a band with the same molecular weight as the positive control (about 500bp) appears in the sample to be tested, it means that the sample is contaminated with mycoplasma. If there is no corresponding band, it means that the sample is not contaminated with mycoplasma.

The electrophoresis image are shown in the figure below:

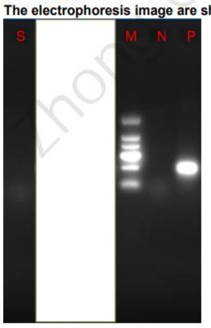

Lane S is SV-HUC-1
Lane M is DNA Marker
Lane N is Negative Control
Lane P is Positive Control

(Note: The band at the bottom of the sample loading wells in the gel image is the primer dimer, which is a common phenomenon in PCR detection. Its intensity is usually inversely proportional to the brightness of the positive band. The PCR detection method is very sensitive, and even a very weak positivity can be distinguished from the dimer, making the results reliable.)

3. Test result

Sample Name	Test Result	Test Criteria	Test Interpretation
SV-HUC-1	-	Compare the positive control to see if there is characteristic band	Negative, no mycoplasma contamination

Shanghai Zhong Qiao Xin Zhou Biotechnology Co.,Ltd.
Reporter: Ke Li
Date: 2024-06-03

上海中乔新舟生物科技有限公司 质检专用章

Zhong Qiao Xin Zhou
