# Supplementary material for: Combined Inhibition of PI3K and STAT3 signaling effectively inhibits bladder cancer growth
Source: Oncogenesis. 2024 Jul 27;13(1):29. doi: 10.1038/s41389-024-00529-y (PMC11283499; doi:10.1038/s41389-024-00529-y)
Supplement: Supplementary file 5 — Supplementary Table 8 [file 41389_2024_529_MOESM5_ESM.pdf]

Drug	Target
Flibanserin	GPCR/G Protein; Neuroscience
Lurasidone	GPCR/G Protein; Neuroscience
Tralkoxydim	Metabolism
Violanthin	Neuroscience
Preladenant	GPCR/G Protein; Neuroscience
Istradefylline	GPCR/G Protein; Neuroscience
Ipatasertib	Cytoskeletal Signaling; PI3K/Akt/mTOR signaling
Uprosertib	Cytoskeletal Signaling; PI3K/Akt/mTOR signaling
Borussertib	Cytoskeletal Signaling; PI3K/Akt/mTOR signaling
Perifosine	Cytoskeletal Signaling; PI3K/Akt/mTOR signaling
ASP3026	Angiogenesis; Tyrosine Kinase/Adaptors; Apoptosis
NVP-TAE 684	Angiogenesis; Tyrosine Kinase/Adaptors; Apoptosis
Mogroside V	PI3K/Akt/mTOR signaling; Chromatin/Epigenetic
HTH-01-015	PI3K/Akt/mTOR signaling; Chromatin/Epigenetic
Darolutamide	Endocrinology/Hormones
Acetohydroxamic acid	Microbiology/virology; Proteases/Proteasome
STM2457	Apoptosis
Gemcitabine	Cell Cycle/Checkpoint; DNA Damage/DNA Repair
Simvastatin	Apoptosis; Autophagy; Metabolism
Bardoxolone Methyl	Apoptosis; Autophagy; NF-κB; Immunology/Inflammation
Mocetinostat	Autophagy; Chromatin/Epigenetic; DNA Damage/DNA Repair
Trametinib	Apoptosis; Autophagy; MAPK
Everolimus	Apoptosis; Autophagy; PI3K/Akt/mTOR signaling
Enzastaurin	Autophagy; Chromatin/Epigenetic; Cytoskeletal Signaling
Verteporfin	Angiogenesis; Apoptosis; Autophagy; Stem Cells

BAI1	Apoptosis
Unesbulin	Apoptosis; Cell Cycle/Checkpoint; DNA Damage/DNA Repair
PTC-028	Apoptosis; Cell Cycle/Checkpoint; DNA Damage/DNA Repair
Dinaciclib	Apoptosis; Cell Cycle/Checkpoint
MYCMI-6	Apoptosis; Cell Cycle/Checkpoint
Erdafitinib	Angiogenesis; Tyrosine Kinase/Adaptors; Apoptosis
Infigratinib	Angiogenesis; Tyrosine Kinase/Adaptors; Apoptosis
Myrislignan	Apoptosis; NF-κB
Dauricine	Apoptosis; NF-κB
BMS-202	Apoptosis; Cell Cycle/Checkpoint;
BMS-1	Cell Cycle/Checkpoint; Immunology/Inflammation
Copanlisib	Apoptosis; PI3K/Akt/mTOR signaling
Buparlisib	Apoptosis; PI3K/Akt/mTOR signaling
iCRT3	Apoptosis; Cytoskeletal Signaling; Stem Cells
PNU-74654	Apoptosis; Cytoskeletal Signaling; Stem Cells
Aurora kinase inhibitor-3	Cell Cycle/Checkpoint; Chromatin/Epigenetic
Aurora kinase inhibitor-2	Cell Cycle/Checkpoint; Chromatin/Epigenetic
Aumitin	Autophagy
MDK-6983	Autophagy
Regorafenib	Angiogenesis; Tyrosine Kinase/Adaptors; Apoptosis; Autophagy; MAPK
Lapatinib	JAK/STAT signaling; Tyrosine Kinase/Adaptors; Apoptosis; Autophagy
AS1842856	Apoptosis; Autophagy
TOMATIDINE HYDROCHLORIDE	Autophagy; MAPK; NF-κB
Glycycoumarin	Autophagy; MAPK; Metabolism
Olaparib	Autophagy; Chromatin/Epigenetic; DNA Damage/DNA Repair
Veliparib	Autophagy; Chromatin/Epigenetic; DNA Damage/DNA Repair
Idelalisib	Autophagy; PI3K/Akt/mTOR signaling

Maritoclax	Apoptosis
MIM1	Apoptosis
Agerafenib	Angiogenesis; Cytoskeletal Signaling; Tyrosine Kinase/Adaptors; Apoptosis; MAPK
Acalabrutinib	Angiogenesis; Tyrosine Kinase/Adaptors
evobrutinib	Angiogenesis; Tyrosine Kinase/Adaptors
HJC0350	GPCR/G Protein
PF-06928215	Immunology/Inflammation
G140	Immunology/Inflammation
M4205	Tyrosine Kinase/Adaptors
Valdecoxib	Immunology/Inflammation; Neuroscience
Celecoxib	Immunology/Inflammation; Neuroscience
CID 5951923	Cell Cycle/Checkpoint;DNA Damage/DNA Repair
SR15006	Cell Cycle/Checkpoint;DNA Damage/DNA Repair
IBR2	Cell Cycle/Checkpoint; DNA Damage/DNA Repair
Osimertinib	JAK/STAT signaling; Tyrosine Kinase/Adaptors
Zorifertinib	JAK/STAT signaling; Tyrosine Kinase/Adaptors
MTX-211	Angiogenesis; JAK/STAT signaling; Tyrosine Kinase/Adaptors; PI3K/Akt/mTOR signaling
Ulixertinib	MAPK
Tizaterkib	MAPK
Futibatinib	Angiogenesis; Tyrosine Kinase/Adaptors
Pemigatinib	Angiogenesis; Tyrosine Kinase/Adaptors
Cannabidivarin	Membrane transporter/Ion channel; Neuroscience
Saclofen	Membrane transporter/Ion channel; Neuroscience
Terphenyllin	Metabolism
Voglibose	Metabolism
AH-7614	Endocrinology/Hormones; GPCR/G Protein
MS21570	Endocrinology/Hormones; GPCR/G Protein
AZD1080	PI3K/Akt/mTOR signaling; Stem Cells

LY2090314	PI3K/Akt/mTOR signaling; Stem Cells
Tucidinostat	Chromatin/Epigenetic; DNA Damage/DNA Repair
Abexinostat	Chromatin/Epigenetic; DNA Damage/DNA Repair
Dynarrestin	GPCR/G Protein; Stem Cells
Ciliobrevin A	GPCR/G Protein; Stem Cells
Belzutifan	Angiogenesis; Chromatin/Epigenetic
Adaptaquin	Angiogenesis;Chromatin/Epigenetic
Desidustat	Angiogenesis; Chromatin/Epigenetic; Metabolism
Betahistine EP Impurity C	Immunology/Inflammation; Neuroscience
Bepotastine	Immunology/Inflammation; Neuroscience
GSK-J1	Chromatin/Epigenetic
JQKD82 trihydrochloride	Chromatin/Epigenetic
EBI-2511	Chromatin/Epigenetic
MM-102	Chromatin/Epigenetic
TC-E 5003	Chromatin/Epigenetic
Picropodophyllotoxin	Tyrosine Kinase/Adaptors
Linsitinib	Tyrosine Kinase/Adaptors
Pimodivir	Microbiology/virology
Tirofiban	Cytoskeletal Signaling
IMD-0560	NF-κB
IMD-0354	NF-κB
ML-226	Metabolism
KT182	Metabolism
Marimastat	Proteases/Proteasome
Batimastat	Proteases/Proteasome
Zotarolimus	PI3K/Akt/mTOR signaling
KU-0063794	PI3K/Akt/mTOR signaling
PQR620	PI3K/Akt/mTOR signaling
Ergolide	NF-κB
Quinoclamine	NF-κB
UZH2	Others
Benzonitrile, 3,3'-[(2-oxo-1,3-cyclohexanediylidene)dimethylidyne]bis-(9CI)	Apoptosis
Talazoparib	Chromatin/Epigenetic; DNA

	Damage/DNA Repair
BMS-1166	Cell Cycle/Checkpoint; Immunology/Inflammation
BMS-1001	Cell Cycle/Checkpoint; Immunology/Inflammation
Olprinone	Metabolism
Enoximone	Metabolism
Seralutinib	Angiogenesis; Tyrosine Kinase/Adaptors
Tariquidar	Membrane transporter/Ion channel; Neuroscience
Duvelisib	PI3K/Akt/mTOR signaling
Alpelisib	PI3K/Akt/mTOR signaling
Eganelisib	PI3K/Akt/mTOR signaling
Tenalisib	PI3K/Akt/mTOR signaling
Umbralisib	PI3K/Akt/mTOR signaling
Darovasertib	Chromatin/Epigenetic; Cytoskeletal Signaling
Midostaurin	Chromatin/Epigenetic; Cytoskeletal Signaling
BpV(HOpic)	PI3K/Akt/mTOR signaling
Dabrafenib	MAPK
Rasarfin	GPCR/G Protein; MAPK
Allopurinol Sodium	Immunology/Inflammation
YKL-05-099	PI3K/Akt/mTOR signaling
HG-9-91-01	PI3K/Akt/mTOR signaling
WAY-303290	Angiogenesis; Tyrosine Kinase/Adaptors
RK-24466	Angiogenesis; Tyrosine Kinase/Adaptors
C-176	Immunology/Inflammation
H-151	Immunology/Inflammation
Entospletinib	Angiogenesis; Tyrosine Kinase/Adaptors
Cevidoplenib dimesylate hydrochloride	Angiogenesis; Tyrosine Kinase/Adaptors
Galunisertib	Stem Cells
Afimetoran	Immunology/Inflammation
Roquinimex	Apoptosis
Isobarbaloin	Proteases/Proteasome
Arbutin	Proteases/Proteasome
Monobenzone	Proteases/Proteasome
Fruquintinib	Angiogenesis; Tyrosine Kinase/Adaptors

Acrizanib	Angiogenesis;Tyrosine Kinase/Adaptors
Adavivint	Cytoskeletal Signaling; Stem Cells
Tegatrabetan	Cytoskeletal Signaling; Stem Cells
Lats-IN-1	Stem Cells
MYF-01-37	Stem Cells
